# Nutrient coverage of China’s plant-based food supply can be improved with food system adjustments

**DOI:** 10.1038/s43016-026-01349-6

**Published:** 2026-05-01

**Authors:** Yijun Li, Johanna C. Gerdessen, Tjeerd Jan Stomph, Anneleen Kuijsten, Sander de Leeuw, Zhiyao Chang, Shenggen Fan, Wen-Feng Cong, Wopke van der Werf

**Affiliations:** 1 Operations Research and Logistics, Wageningen University and Research, Wageningen, The Netherlands; 2 College of Resources and Environmental Sciences, State Key Laboratory of Nutrient Use and Management, China Agricultural University, Beijing, China; 3 Academy of Global Food Economics and Policy, China Agricultural University, Beijing, China; 4 Centre for Crop Systems Analysis, Wageningen University and Research, Wageningen, The Netherlands; 5 Division of Human Nutrition and Health, Wageningen University and Research, Wageningen, The Netherlands; 6 College of Food Science and Nutritional Engineering, China Agricultural University, Beijing, China

**Keywords:** Agriculture, Nutrition

## Abstract

Plant-based foods provide most nutrients for human diets and have wide availability, yet nutrient gaps persist in many regions. Here we assessed China’s plant-based food supply during 1997–2018 to determine its ability to meet population-level requirements for dietary energy and 17 nutrients and evaluated food system scenarios for improving nutrient coverage and source diversity. In the baseline scenario including animal-based foods, coverage was high for energy, protein, carbohydrates, dietary fibre and ten micronutrients; medium for riboflavin and vitamin A; and low for calcium and selenium. Halving food loss and waste, increasing whole-grain use and moderating red meat intake improved coverage for all nutrients by 8–44% above baseline. Redirecting crops from feed and non-food uses to human consumption enabled self-sufficiency for energy and 14 nutrients. While food system improvements in China can strengthen nutrient availability, complementary strategies, such as crop diversification, fortification and biofortification are needed to close the remaining gaps.

## Abstract (web summary)

China’s plant-based food supply provides much of the population’s energy and nutrient needs, but key micronutrient gaps persist. Nutrient coverage can be strengthened by reducing food loss and waste, increasing whole-grain use, moderating red meat intake and repurposing crops from feed and non-food uses to direct human consumption.

## Main

Food and nutrition security depend on food systems that supply sufficient amounts of all nutrients from diverse sources^1,2^. However, the current food systems face challenges including unbalanced diets (for example, high in meat and low in whole grains and fruits), malnutrition and environmental externalities (for example, greenhouse gas emissions, overuse of water)^1,3^. Increasing the share of plant-based foods may reduce environmental externalities while supporting healthier diets for balanced nutrition^3,4^. Assuring a supply that matches population requirements is a precondition for sufficient daily intake of all nutrients. Diversity of crops supplying nutrients could contribute to the robustness of the food supply system^2^ and support tackling micronutrient deficiencies^5^. Therefore, it is essential to assess the capacity of plant-based foods to supply nutrients in a sufficient and diverse way, supporting nutrition security and environmental sustainability.

Currently, the nutrients provided from plant-based foods are not used efficiently: direct nutrient losses occur via food loss and waste^6^ and refinement of grains^7^ and indirect losses occur when crops are used for animal feed or non-food uses (industrial use) instead of direct human consumption^8,9^. Modelling approaches can help link nutrient availability to population-level needs and identify strategies that ensure a sufficient and diverse supply of nutrients. Recent modelling studies assessed whether global nutrient availability from food sources can meet population-level requirements for up to 11 nutrients, accounting for production, trade, food loss, waste and inedible parts to estimate nutrient availability, and these reported worldwide shortfalls for micronutrients such as calcium and vitamin A^10,11^.

For supply diversity, a global analysis of crop–nutrient supply networks reported a positive yet levelling-off relationship between the number of crops supplied and their ability to meet nutrient intake under perturbations (for example, production failures from drought or import failures due to trade war)^2^. However, the study measured the ability by assessing the mere presence of nutrients in the supplied crops rather than the quantity of nutrients available for consumption and thus could not capture the extent to which nutrient needs were met^2^.

To account for local differences in food sources and formulate context-specific strategies, investigations for a specific country are required, as countries and subnational regions differ in import dependence, diets, prevalence of insufficient nutrient intake and nutrient deficiencies^2,11,12^. Understanding whether a country’s plant-based food supply can meet population-level nutrient needs and, if so, how this could be achieved via potential strategies is important for informing policies aiming to achieve national nutrition security while advancing sustainability.

China is home to 22% of the world’s population and plays a significant role in the production, import, and export of numerous food commodities^13,14^. Traditional Chinese diets are largely plant based, but rising red meat consumption in recent decades has increased reliance on locally produced or imported feed for meat production^15–17^. Despite potential resilience benefits from international trade, this growing dependency has increased environmental externalities in China and feed-exporting countries alike^17–19^. Studies based on nutrient intake from diets indicate that among the Chinese population, the intake of energy and macronutrients (carbohydrates, protein, fats) generally meets the intake needs^20^. However, for some micronutrients, a subset of the population failed to meet recommended intakes and showed no improvement from 2010 to 2017, and dietary fibre intake has declined over the same period^20–23^. Nutrient status from biomarker evidence indicates a prevalence of insufficiency or deficiency in iron, zinc, selenium and vitamins A and D in some age groups during 2010–2019^24,25^. It is therefore important to analyse the capacity of plant-based foods to supply nutrients in a sufficient and diverse way and explore options for improving micronutrient provisioning through plant-based foods.

We define plant-based food supply (PFS) as the supply of plant-based foods for human consumption through multiple supply chain stages, accounting for domestic production, international trade, non-food use, food loss and waste and inedible parts. We consider 30 crop sources from six crop groups (cereals, tubers, beans, fruits, vegetables, oil crops), together with animal-based foods, for the provisioning of dietary energy and 17 nutrients. Because sufficient vitamin D status is difficult to achieve through diets alone^26^, and vitamin B12 is essentially only provided by animal-based foods^27^, these two nutrients are not included in our main analysis. For each nutrient, we quantify two dimensionless indicators: coverage (the supply/demand ratio) and source diversity, measured as the exponentiated Shannon entropy of proportional supply from different crops and animal-based foods (the latter as a single group)^28^. We assess trends in coverage and diversity from plant-based foods during 1997–2018 and conduct scenario analyses to evaluate the impact of food system transformations on the sufficiency and diversity of each nutrient supply (Table 1). Scenario S1 (Baseline) assesses nutrient supply from plant- and animal-based foods in 2018. Scenario S2 evaluates more efficient nutrient use from PFS by reducing direct and indirect nutrient losses. Scenario S3 examines nutrient adequacy when all domestically produced crops are used for direct human consumption, without animal-based foods and international trade, at the national level with inter-provincial trade (S3a) and at the provincial level without such trade (S3b).

**Table 1:** Food system adjustment scenarios

	Scenario S1 Baseline	Scenario S2 Reducing nutrient losses		Scenario S3 Self-sufficiency	
		S2a Reducing direct nutrient losses	S2b Reducing indirect nutrient losses by reducing red meat intake	S3a National	S3b Provincial
Aggregation level	National	National	National	National	Provincial
Food loss and waste	Current	−50%	−50%	−50%	−50%
Replacement of refined grains	Current	+10%	+10%	+10%	+10%
Intake of animal-based food sources	Current	Current	45% less red meat	No	No
Feed use of food crops	Current	Current	Reduced according to red meat reduction	No	No
Non-food use of food crops	Current	Current	Current	No	No
International trade	Current	Current	Current	No	No
Inter-provincial trade	Yes	Yes	Yes	Yes	No

Five scenarios for adjusting China’s plant-based food supply were examined, differing in aggregation level (national or provincial), food loss and waste (current or reduced by 50%); replacement of refined grains with whole-grain alternatives (current or 10% replaced); intake of animal-based food (current or according to recommendations, which means 45% reduction of red meat intake, or zero), resulting in a repurposing of feed crops for human consumption (none, partial or full); non-food use of food crops (current or none); international trade (current or none) and inter-provincial trade (current or none). Other attributes, such as crop production and seed use amount, were the same in all scenarios.

## Results

### Scenario S1 baseline coverage and source diversity

We used two widely used metrics to assess the fulfilment of nutrient needs in the population: (1) estimated average requirement (EAR), that is, the level of intake that meets the needs of 50% of all individuals in an age- and sex-specific stratum of the population and (2) recommended nutrient intake (RNI), that is, the level of intake that meets the needs of 97.5% of individuals in each stratum^29,30^. RNI is set higher than EAR and coverage of RNI thus confers greater certainty that supply is sufficient. For potassium, we used adequate intake (AI), and for energy, estimated energy requirement (EER) as nutrient needs. Coverage was classified as high (supply ≥ RNI), medium (EAR ≤ supply < RNI) or low (supply < EAR) for those nutrients for which EAR and RNI are defined. For potassium and energy, we considered supply above AI or EER as high, 80–100% as medium and below 80% of AI or EER as low. We compared source diversity among nutrients but did not classify it as high/medium/low because there is no literature proposing a threshold of source diversity to ensure a robust supply.

**Fig. 1:**
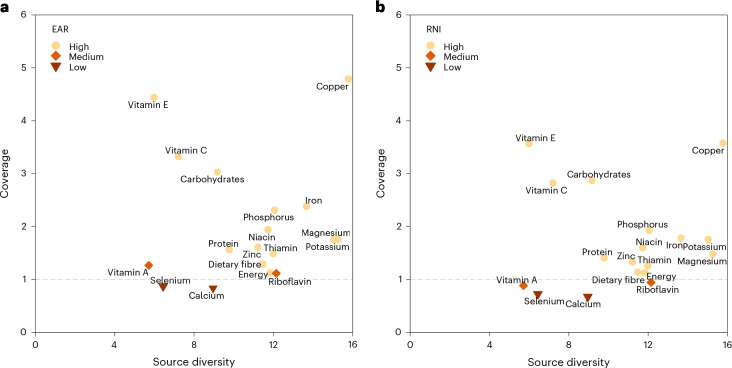
Coverage and source diversity of 17 nutrients and dietary energy from plant- and animal-based food sources in Scenario S1 baseline. **a**,**b**, Coverage relative to EAR versus source diversity (**a**) and coverage relative to RNI versus source diversity (**b**). Both coverage (supply/demand ratio) and source diversity (exponentiated Shannon entropy of proportional supply from different crops and animal-based foods) are dimensionless numbers. Each nutrient’s coverage was classified as high (supply ≥ RNI), medium (EAR ≤ supply < RNI) or low (supply < EAR). For potassium and energy, only AI and EER are available; we classify coverage below 80% of AI or EER is low, 80–100% is medium and above AI or EER is high. Source diversity was compared across different nutrients. A higher source diversity for a nutrient indicates that compared with a nutrient with lower source diversity, it has a more diverse and balanced supply of crop sources. Source data

In the baseline Scenario S1, coverage was high for energy, protein, carbohydrates, dietary fibre and ten micronutrients, medium for riboflavin and vitamin A, while low for calcium and selenium (Fig. 1). The source diversity of nutrients ranged from 5 to 16, which may be interpreted as the demand for those nutrients being effectively supplied by 5 to 16 equally important crop sources^28^. Copper, magnesium and potassium had the highest source diversity ( > 15), vitamins A, E and selenium had the lowest ( < 7), while the other nutrients had intermediate source diversity (7–15).

### PFS contributions to baseline coverage and temporal changes in coverage and source diversity

**Fig. 2:**
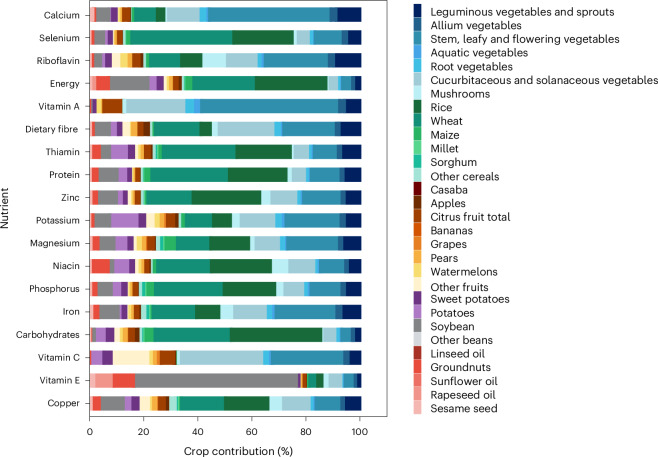
Percent contribution of different crops and crop groups to the total plant-based coverage of dietary energy and nutrient needs of the Chinese population under Scenario S1 baseline. This percentage does not depend on whether the estimated average requirement or the recommend nutrient intake is used as a reference intake. Source data

**Extended Data Fig. 1:**
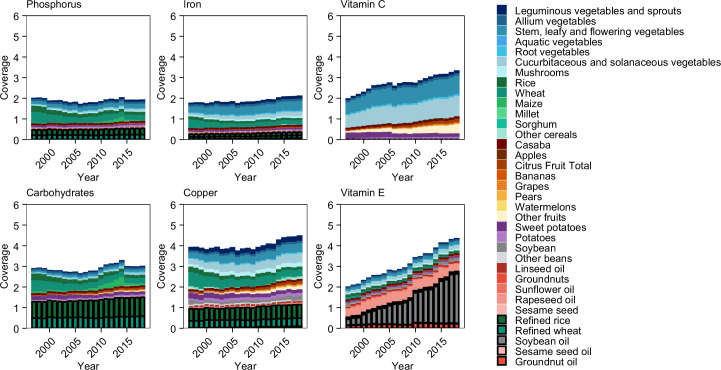
Coverage of six nutrients from plant-based foods in the baseline scenario (excluding animal-based food supply) during 1997-2018. Coverage (supply/demand ratio) was calculated using the adequate intake for potassium, the estimated energy requirement for energy, and the estimated average requirement for the other nutrients. Calculations for 1997-2009 and from 2010 onward were based on FAOSTAT Food Balance Sheets using the “old” and “new” methodologies, respectively. Coverage from oil crops was separated in the part provided by the oil and directly by the seeds, as appropriate. Source data

**Extended Data Fig. 2:**
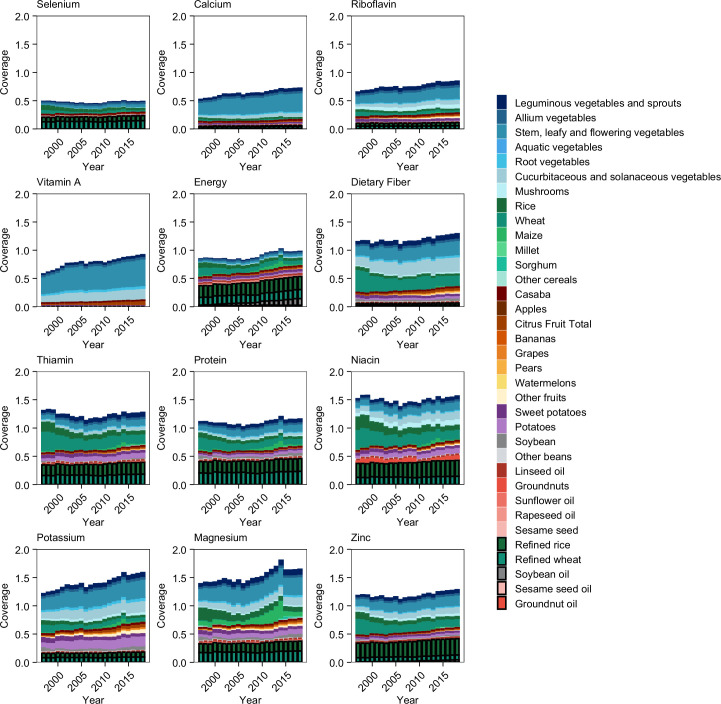
Coverage of dietary energy and 11 nutrients from plant-based foods in the baseline scenario (excluding animal-based food supply) during 1997-2018. Coverage (supply/demand ratio) was calculated using the adequate intake for potassium, the estimated energy requirement for energy, and the estimated average requirement for the other nutrients. Calculations for 1997-2009 and 2010 onward were based on FAOSTAT Food Balance Sheets using the “old” and “new” methodologies, respectively. Coverage from oil crops was separated in the parts provided by the oil and directly by the seeds, as appropriate. Source data

In Scenario S1, the contribution of PFS to coverage ranged from 56% for selenium to over 98% for vitamin C, vitamin E, carbohydrates and dietary fibre (Supplementary Table 1). Within PFS, vitamin E was mainly supplied by vegetable oil and vegetables (for example, cucurbitaceous and solanaceous and stem, leafy and flower vegetables) (Fig. 2 and Extended Data Fig. 1). Cereals and vegetable subgroups were the main sources of coverage for other nutrients. Among cereals, major staples (that is, wheat and rice) contributed more to all nutrients than more nutrient-dense crops such as millet, sorghum and other cereals, primarily due to the higher production volumes of the major staples (Fig. 2). Soybean and sesame contributed little to coverage of most nutrients because most of their supply for food consumption was processed into vegetable oil (Fig. 2 and Extended Data Figs. 1 and 2). Other nutrient-dense beans also contributed little to coverage, due to low production.

**Extended Data Fig. 3:**
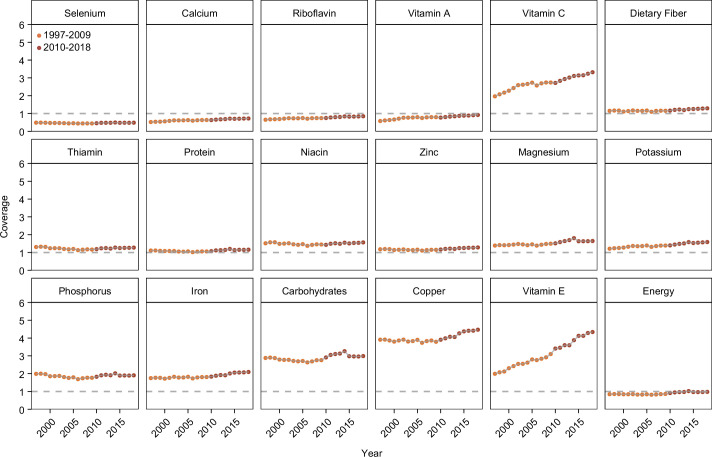
Temporal trend of coverage for 17 nutrients and dietary energy in China between 1997 and 2018, based on plant-based food supply (excluding animal-based supply) in Scenario S1 (Baseline). Coverage (supply/demand ratio) was calculated using the adequate intake for potassium, the estimated energy requirement for energy, and the estimated average requirement for the other nutrients. Calculations for 1997-2009 and from 2010 onward were based on FAOSTAT Food Balance Sheets using the “old” and “new” methodologies, respectively. Source data

**Extended Data Fig. 4:**
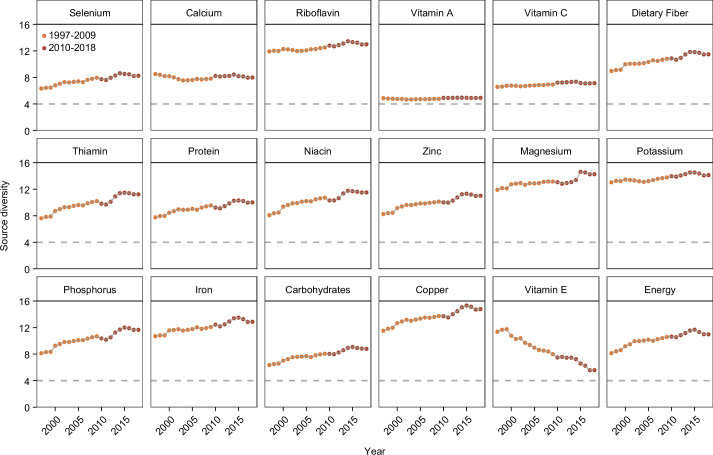
Temporal trend of source diversity for 17 nutrients and dietary energy in China between 1997 and 2018, based on plant-based food supply (excluding animal-based supply) in Scenario S1 (Baseline). Calculations for 1997-2009 and from 2010 onward were based on FAOSTAT Food Balance Sheets using the “old” and “new” methodologies, respectively. Source data

Over time (1997 to 2018), minimal improvement in coverage occurred from PFS for nutrients with medium or low coverage, that is, riboflavin, vitamin A, selenium and calcium, while the source diversity of vitamin E decreased from 11 to 6 due to the increasing contribution from vegetable oil (Extended Data Figs. 3 and 4).

### Scenario S2 using nutrients more efficiently by reducing nutrient losses

**Fig. 3:**
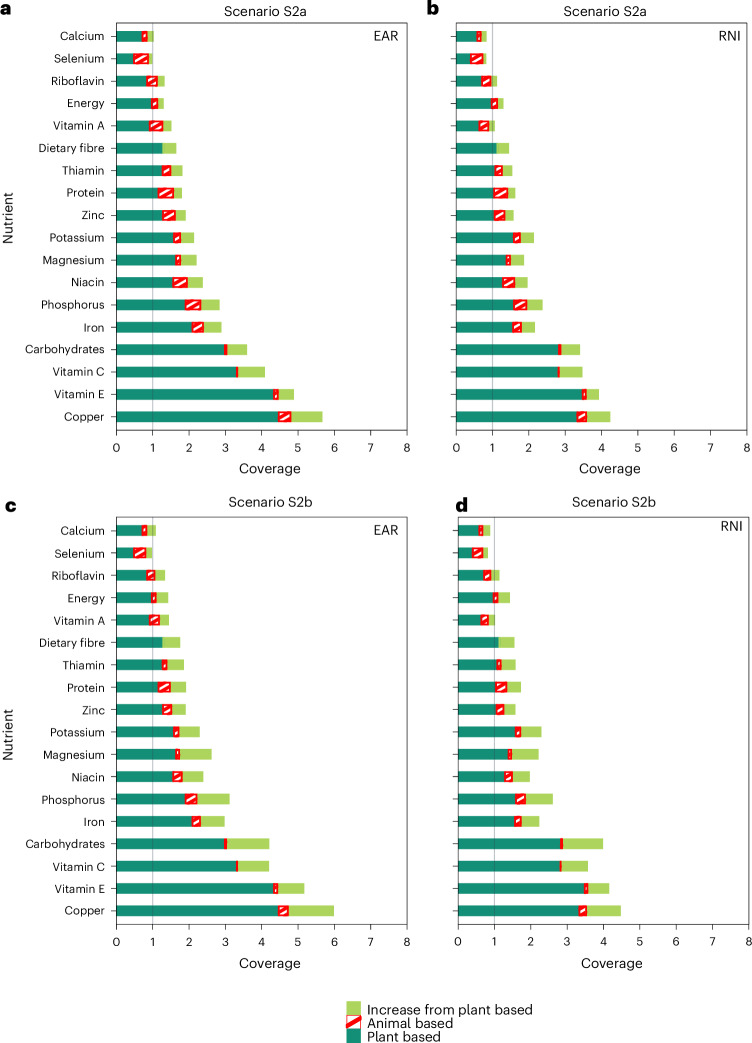
Coverage from plant- and animal-based food sources and increase in coverage from plant-based food supply compared to baseline. **a**,**b**, Scenario S2a (reducing direct nutrient losses). **c**,**d**, Scenario S2b (in addition, reducing indirect nutrient losses by reducing red meat intake and repurposing feed for food use). From panels **a** to **c** and **b** to **d**, coverage from animal-based sources slightly decreased due to reduced consumption. For most nutrients and dietary energy, this reduction is more than offset by an increase from plant-based sources. Coverage (supply/demand ratio) is calculated using EAR (**a**,**c**) or RNI (**b**,**d**) as reference intake. Source data

**Extended Data Fig. 5:**
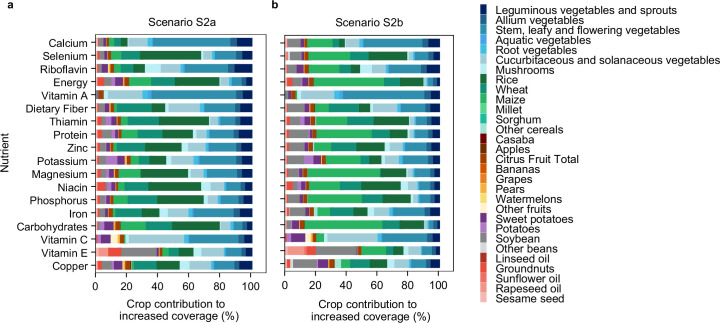
**The percentage contribution of each plant-based nutrient source (%) to the total coverage increase under Scenarios S2a (Panel a) and S2b (panel b) relative to the baseline**. Source data

Coverage of energy and 17 nutrients was increased by an average 22% (range: 8 to 44%) compared to the baseline when halving food loss and waste, increasing whole-grain use (Scenario S2a) and reducing red meat intake (Scenario S2b) (Fig. 3 and Extended Data Table 1). Source diversity was improved by an average 5% (range: −9% to 33%) (Extended Data Table 1). Gains in source diversity from Scenarios S2a and S2b were modest because most of the increased nutrient supply came from crops that already contributed substantially to the baseline coverage, such as rice, wheat and stem, leafy and flower vegetables (Extended Data Fig. 5).

Overall, calcium coverage improved from low to medium and riboflavin coverage improved from medium to high if nutrient losses were reduced (Fig. 3). Selenium coverage, however, remained low and the source diversity of vitamin A ( < 6) remained the lowest among all nutrients (Extended Data Table 1).

### Scenario 3 self-sufficiency

**Fig. 4:**
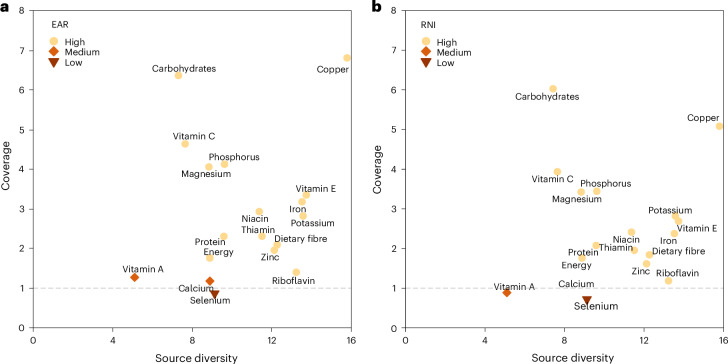
Coverage and source diversity of dietary energy and 17 nutrients under Scenario S3a national self-sufficiency. Scenario S3a analysed which level of self-sufficiency could be reached when all domestically produced food crops would be used for direct human consumption and international trade and the intake of animal-based food were absent. **a**, Coverage relative to EAR versus source diversity. **b**, Coverage relative to RNI versus source diversity. Both coverage (supply/demand ratio) and source diversity (exponentiated Shannon entropy of proportional supply from different food sources) are dimensionless numbers. Nutrient coverage was classified as high (supply ≥ RNI), medium (EAR ≤ supply < RNI) or low (supply < EAR). For potassium and energy, coverage was classified using AI and EER: <80% as low, 80–100% as medium and >100% as high. Higher source diversity of a nutrient indicates that the nutrient has a more diverse and balanced supply of sources than other nutrients with lower source diversity. Source data

Making all domestically harvested food crops (including maize and soybean currently used for non-food) available for direct human consumption (Scenario S3a) increased the national coverage of dietary energy needs to 1.8 even if international trade was absent, assuming the efficiency measures of Scenario S2a were also applied (Fig. 4 and Extended Data Table 1). However, the coverage of calcium, selenium and vitamin A remained below the RNI. As a result, China is not self-sufficient in terms of micronutrient provision in Scenario S3a.

**Fig. 5:**
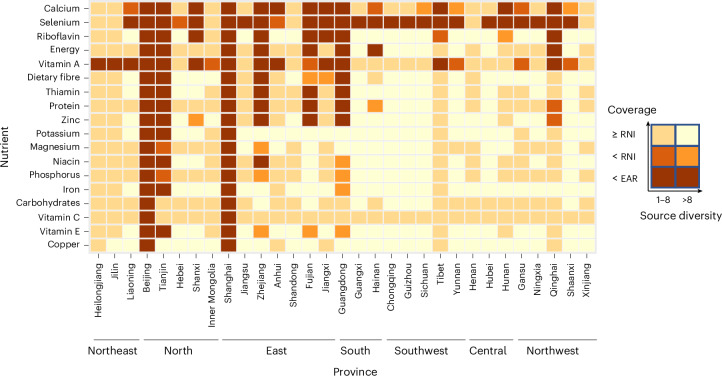
Coverage and source diversity of dietary energy and 17 nutrients under Scenario S3b provincial self-sufficiency. Scenario S3b aimed to analyse which level of self-sufficiency per province could be reached when all domestically produced food crops would be used for direct human consumption and international and inter-provincial trade is absent. Coverage (supply/demand ratio) was calculated using the adequate intake for potassium, the estimated energy requirement for energy and the estimated average requirement (EAR) and recommended nutrient intake (RNI) for the other nutrients. Provinces are shown in seven regions: northeast, north, east, south, southwest, central and northwest in China^81^. Source data

Scenario S3b assumed the absence of international and inter-provincial trade to evaluate the spatial heterogeneity of PFS at the provincial level and assess risks of reliance on external supplies. In more than 20 of the 31 provinces, coverage from PFS was high for energy and all nutrients except calcium, selenium and vitamin A (Fig. 5). The coverage of selenium and calcium was low in 23 and 12 provinces, respectively. Highly urbanized provinces such as Beijing, Shanghai and parts of East China had low coverage for most nutrients, with coverage for calcium and vitamin A dropping to as low as 0.2 on average (based on EAR) in Beijing and Shanghai, highlighting the critical role of logistics in balancing supply and consumption and the dependency of urban regions on food supply from outside.

## Discussion

The baseline supply of plant-based and animal-based food sources (Scenario S1) fulfilled the needs for energy and 13 nutrients, but it did not sufficiently meet the population’s needs for calcium, selenium, riboflavin and vitamin A. Vitamins A and E and selenium had lower source diversity than other nutrients. Tracking coverage and source diversity per nutrient from plant-based foods from 1997 to 2018 showed little improvement in coverage for calcium, selenium, riboflavin and vitamin A and in source diversity of vitamins A and E. Halving food loss and waste, increasing whole-grain use (Scenario S2a) and moderating red meat intake (Scenario S2b) raised coverage of all nutrients by 8–44% relative to the baseline but had smaller and varied effects on source diversity. If all domestically produced food crops would be available for human consumption without international trade (Scenario S3a), PFS would meet the population’s needs for dietary energy and 14 nutrients, but it would remain insufficient for selenium, calcium and vitamin A at the national level.

Data from previous studies on nutrient intake from diets and nutrient status from biomarker evidence provide context to these results. Nutrient supply in the baseline and reported nutrient intake were sufficient for energy and macronutrients (protein, carbohydrates)^20^, and for copper (only 2.6% of adults had insufficient intake)^23^, indicating that the current food system and dietary patterns effectively support these nutrients. We found shortages in the supply of calcium, selenium, riboflavin and vitamin A. These findings are in line with evidence from consumption data^20,21,23^ and nutrient status^24,25^. Large proportions of the Chinese population had insufficient intake of calcium, selenium, riboflavin and vitamin A from 2010 to 2017^20,21,23^, while nutrient status evidence shows deficiencies in selenium among children^25^, marginal vitamin A deficiency in children/adolescents (15%) and in adults (4%) during 2012–2017^24^ (Supplementary Tables 2 and 3). Nationwide biomarker evidence of calcium and riboflavin after 2010 was not found.

Our results demonstrate a mismatch between the modelled nutrient supply and earlier reported nutrient intake and/or nutrient status data. The baseline supply (2018) for thiamin, potassium, magnesium, vitamin C and dietary fibre was sufficient, but inadequate intake was reported during 2010–2017 (Supplementary Table 2)^20,21,23^. For niacin, phosphorus and vitamin E, baseline supply was sufficient, but 21–39% of adults still had inadequate intake^23^. National nutrient status information for these nutrients was not available after 2010. The supply of iron and zinc was sufficient and diverse, while 12% and 36% of adults had inadequate intake of iron and zinc^20^, and nutrient status evidence reported deficiencies in several groups (Supplementary Tables 2 and 3)^24,31^. The mismatch between sufficient supply and insufficient intake can be explained by factors not fully captured in our modelling, such as the distribution of foods to different regions and subsections of the population. There may be a problem of access, as discussed below. A possible further factor driving differences between supply and nutrient deficiency based on biomarker evidence is differences in bioavailability. The bioavailability of any particular micronutrient highly depends on food sources, dietary combinations, inhibitors (for example, phytates), enhancers (for example, vitamin C) and individual differences^32,33^. Consequently, even if national-level supply is sufficient, different diets across regions and population subgroups can lead to variation in bioavailability and, ultimately, nutrient status^12^.

Rice and wheat contributed most to the coverage of dietary energy in the baseline scenario (S1). Reducing nutrient losses (Scenario S2) and repurposing domestically harvested crops for direct human consumption (Scenario S3a) increased dietary energy supply to at least 1.4 times requirement. This surplus creates opportunities to shift from rice and wheat to more nutrient-dense crops that may have lower yields but higher micronutrient production per hectare and require lower water and fertilizer input^34^ (Supplementary Table 4). Such a shift could benefit public health and planetary health but would require redesigning food systems to support healthy diets that are nutritionally sufficient and diverse and policies that ensure economic viability for stakeholders^35^ and encourage dietary change.

Scenario S3b (provincial self-sufficiency) highlights the risks of uneven food distribution across different provinces and the potential problem of access: some regions, particularly highly urbanized regions, remain dependent on external supplies (Fig. 5). Although distribution disruptions are rare, their impact on food delivery and nutrition security^36,37^ underscores the importance of emergency supply plans, such as maintaining buffer stocks, strengthening transportation and distribution systems and increasing regional self-sufficiency in essential nutrients.

Selenium coverage was low in all scenarios. Biomarker evidence shows a significant positive correlation between selenium levels in the human body and selenium content in consumed grains^38^. Selenium content in grains is mainly influenced by soil and fertilizers used, so grains can be a good or poor source of selenium^39,40^. Selenium-enriched fertilizers can increase wheat and rice selenium content more than fivefold^41,42^. Finland, for example, increased selenium content of food through the nationwide use of selenium-enriched fertilizers proving this is a feasible way to boost selenium supply from plant-based foods^43^. Also, eggs and aquatic foods, which are rich in selenium^44^ and have lower environmental impacts than other animal-based foods^3,45^, merit further investigation to sustainably meet selenium needs.

Adjusting supply sources of PFS by increasing the share of crops rich in calcium and vitamin A can enhance nutrient supply while benefiting environmental sustainability^34,46,47^. For instance, replacing staples with beans and fresh fruits can enhance vitamin A provisioning and diversify supply sources^34^. In China, black rice, which is marketed as whole grains, could also be considered for enhancing diet quality^48^. Furthermore, biofortification (increasing nutrient content of crops through breeding, transgenic techniques or agronomic practices)^49^, food fortification (adding nutrients to foods, for example, vitamin A-fortified oils and cereal flours) and supplementation (providing vitamins and minerals in concentrated form, such as pills, capsules or syrups, to increase dietary intake) offer practical solutions to enhance nutrient availability and support nutrition security^38,50^.

Given the widespread vitamin D deficiency among Chinese children and adults^24^, coupled with insufficient dietary intake^51^, reducing the supply of animal-based foods, as in Scenario S3, would further limit the availability of vitamin D. As plant-based foods do not contribute to vitamin D, alternative solutions such as adequate UV exposure, use of supplements, food fortification and biofortification are needed to address vitamin D deficiencies^26,52^. Mushrooms have been suggested as a potential source of vitamin D, but they do not provide meaningful vitamin D amounts unless they are UV exposed^53^. Even then, they provide vitamin D2, which in general is less effective than vitamin D3 at increasing vitamin D levels in the human body and may even reduce the effectiveness of vitamin D3^54^. Likewise, reducing animal-based foods lowers vitamin B12 provisioning. Supplementation and food fortification are crucial to prevent potential vitamin B12 deficiency, particularly when diets shift towards more plant-based or vegan patterns^27^.

We based our analyses on all the publicly available supply data for plant- and animal-based foods in China, but data limitations exist. Bioavailability estimates are incorporated into China’s dietary reference intakes at the population level^30^. However, when proposed diets differ substantially from those on which nutrient intake needs were set, the underlying bioavailability assumptions may no longer hold. This is especially relevant in Scenario S3, where there is a substantial reduction in animal-based foods compared to the baseline. When plant-based foods dominate the diet, the bioavailability of iron and zinc may be reduced due to food component interactions^32,33^; as a result, in Scenario S3, the coverage of iron and zinc would decrease. We did not consider regional variations in crop nutrient content due to soils, climate and agricultural practices in the provincial-level analysis due to data limitations.

The production and supply datasets^55,56^ that we used do not distinguish vegetables into specific species. Previous studies treated vegetables as a single group or split them into three subgroups using their production proportions during 1997–2003^11,57^. Here we used consumption shares from the China Health and Nutrition Survey 2011 dataset to disaggregate the total vegetable supply into seven subgroups to better capture the contribution of vegetables to coverage and source diversity. In reality, consumers have potential intake of a great variety of vegetables. The presented estimates of source diversity in our study therefore set a lower bound to the actual diversity and allow a first comparison between nutrients and an assessment of temporal trends (Extended Data Fig. 4). Ultimately, greater disaggregation of food sources, particularly vegetables, in future production and supply datasets, along with information on whether they are typically consumed fresh or cooked in consumption datasets, would allow a more refined assessment of nutrient provisioning.

**Extended Data Fig. 6:**
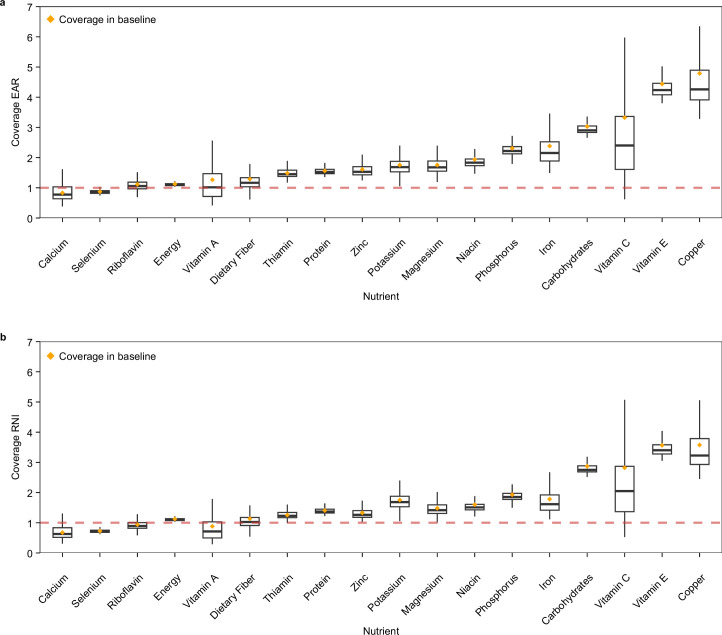
Boxplots illustrating the distribution of coverage for each nutrient in Scenario S1 (Baseline) when analysing the consequences of uncertainty in the detailed composition of supply due to data aggregation. Panel **a** shows coverage relative to estimated average requirement (EAR) while Panel **b** shows coverage relative to recommended nutrient intake (RNI) Both were calculated in Monte Carlo simulations. We used adequate intake as a reference for potassium and estimated energy requirement for energy. We performed 1000-run Monte Carlo simulations (n = 1000) to explore the sensitivity of matching supply datasets with food composition tables, where in each run the nutrient content of one randomly selected member of each food group or crop product was used to represent the nutrient content of that group or crop species. This approach was applied to crops with multiple species (for example, stem, leafy, and flower vegetables, other cereals) or sub-products (for example, white and yellow maize flour). We used the “random.choice” function from Python with a fixed random seed (42) to ensure reproducibility. Each box shows the median, the first and third quartiles from 1000 runs. The whiskers are 1.5 times the interquartile range. The nutrient content for copper is highly skewed (log-normal). Outliers (values outside the whiskers) are not displayed to better visualize the distribution of the data. Source data

Food composition tables report nutrient content for specific species (for example, peppers or squash)^58^, however production datasets report at much lower resolution, that is, for broad groups such as vegetables. This complicates the estimation of nutrient supply. We assessed the resulting uncertainty in nutrient supply using Monte Carlo simulations in which we selected in each run of the supply chain model for each food group the nutrient content of one random member of the food group to calculate coverage. We found that for nutrients with baseline supply below required needs (calcium, selenium, riboflavin, vitamin A), their supply remained below the required needs in over 70% of simulations (Extended Data Fig. 6 and Supplementary Notes).

Food preparation can have a major impact on vitamin content^59^. This is especially relevant for nutrients with medium or low coverage (for example, vitamin A and riboflavin) and nutrients that are sensitive to food preparation (for example, vitamin C). Commonly used methods in China, such as stir-frying, tend to preserve nutrients more effectively than boiling, and using cooking water to make gravy or stock can help retain vitamins when vegetables are cooked. We were unable to exactly quantify the effects of food preparation because of the lack of information on how each food group is typically prepared (fresh, boiled or stir-fried). We did not incorporate the effects of cooking on nutrient provisioning in the baseline scenario. To explore a worst-case scenario, we did a supplementary analysis in which it was assumed that all vegetables are boiled before consumption and the cooking water was not further used. Under this assumption, vitamin A, riboflavin and vitamin C coverage decreased by 6%, 15% and 31%, respectively, relative to the baseline (Supplementary Table 5). Nutrient losses during cooking require attention because baseline coverage of vitamin A and riboflavin was medium. The availability of vitamin C, however, despite potential substantial losses due to cooking, still exceeded population-level needs.

Sufficient and diverse nutrient supply alone does not guarantee healthy, affordable and appealing diets for all. Achieving these outcomes requires food system approaches that consider dimensions such as equitable distribution, appropriate processing and the affordability of nutritious foods^60^. Moreover, although plant-based food supply, together with supplementation and fortification, may support healthy diets, achieving adequate intake and improved nutrient status at the population level depends on other factors including food environments and consumer behaviour. Monitoring micronutrient status is crucial because it provides objective evidence of whether food supply, food environments and diets meet nutrient needs. It links dietary intake to body requirements and absorption, uncovering invisible deficiencies. Such evidence can help guide diet diversification strategies, as nutrient deficiencies vary across populations by age, sex, income and context. Monitoring micronutrient status across China is therefore critical to assess the impacts of the current food system and of policies aimed at transforming it to improve human and planetary health.

Modelling approaches can help to explore how food system adjustments can improve nutrient provisioning and environmental sustainability. Identifying supply–demand nutrition gaps and their causes requires an integrated interpretation of evidence from nutrient supply along the food supply chain, nutrient intake from diets and biomarker-based nutrient status of the population. We integrated this evidence during 2010–2019, identified nutrients that were not effectively supported by current food systems and analysed food system adjustments to improve nutrition outcomes. Our analysis shows that food system adjustments towards improved utilization of plant-based foods can improve nutrient provisioning; however, diversification, biofortification, food fortification and supplementation remain important to meet all nutrient needs, particularly in diets without animal-based foods.

## Methods

### Overview

We evaluated the potential of China’s plant-based food supply (PFS) to meet the population’s needs for energy and 17 nutrients. The plant-based food supply (PFS) was defined as all plant-based foods available for human consumption across the supply chain. To assess the role and potential of PFS to meet the population’s needs for nutrients, we used two dimensionless indicators, coverage (supply-to-demand ratio) and source diversity (exponentiated Shannon entropy). Coverage characterizes the theoretical sufficiency of the total amount supplied to meet the dietary intake needs of the population, while diversity characterizes the variety of sources for each nutrient^2^.

We analysed baseline (Scenario S1) and alternative scenarios (S2 and S3) to explore the potential for improving the coverage and source diversity from plant-based food supply by alterations in the supply chain and the food system relative to the year 2018. We also analysed the temporal trend (1997–2018) of coverage and source diversity per nutrient from plant-based foods at the national level to investigate whether there is any improvement. The study period for the historic trend was selected to uncover trends most relevant to nutrition security and current policy-making while ensuring the use of the most up-to-date crop production data available. Scenario S2a (reducing direct nutrient losses) assumed reduced food loss and waste and a 10% increase in brown rice and whole-wheat flour supply. Scenario S2b (reducing indirect nutrient losses) assumed reduced red meat intake, which frees up crops used for animal feed (for example, maize) as a potential source for direct human consumption or substitution with other crops. Scenario S3 explored the self-sufficiency for each nutrient when all domestically produced crops were used for direct human consumption, animal-based food consumption was reduced to zero and international trade was absent, at the national level with inter-provincial trade (S3a) and at the provincial level without inter-provincial trade (S3b).

### Crops and nutrients considered

We considered 30 crops belonging to six crop groups in our modelling of supply sufficiency and diversity. The six groups were cereals, tubers, beans, fruits, vegetables and oil crops. Cereals comprised five crop species (rice, wheat, maize, sorghum and millet) plus a rest group of other (pseudo)cereals (buckwheat, oats, barley and so on). Tubers comprised potatoes and sweet potatoes. Fruits comprised pears, apples, bananas, all citrus fruit as a single category (oranges, grapefruit and so on^61^), grapes, watermelons, casaba and other fruits (mangoes, berries and so on) (that is, seven categories plus a rest group). Beans comprised soybean and other beans (combination of mung bean, adzuki bean and so on). Oil crops comprised linseed, groundnut, sunflower seed, rapeseed and sesame seed. Vegetables were subdivided into seven subgroups based on their consumption shares^62^: root vegetables (for example, radish); leguminous vegetables and sprouts (for example, soybean sprouts); cucurbitaceous and solanaceous vegetables (for example, eggplant); allium vegetables (for example, scallion); stem, leafy and flower vegetables (for example, cabbage, broccoli); aquatic vegetables (for example, lotus root) and mushrooms (biologically fungi, but often classified within the vegetable group in production information and food grouping systems^55,63^). This subdivision of vegetables into subgroups does not fully represent the diversity of vegetable species consumed, but the available data sources (FAOSTAT^56^ and the National Bureau of Statistics of China^55^) do not allow for including more detail. The considered crops account for more than 95% of Chinese residents’ dietary intake of plant-based foods (based on the China Health and Nutrition Survey 2011^64,65^).

We analysed the supply from these 30 crops and animal-based food sources for dietary energy and 17 nutrients, namely calcium, potassium, magnesium, iron, copper, vitamin A (reported as retinol activity equivalents^58^, which was calculated as retinol plus β-carotene/12 plus other carotenoids/24), riboflavin, vitamin C, vitamin E, phosphorus, zinc, selenium, thiamin, niacin, protein, carbohydrates and dietary fibre. The selected nutrients include those reported to have insufficient intake in the Chinese population^31^ and for which complete nutrient content data is available in the Chinese Food Composition Tables^58,66^. Other nutrients, for example, vitamins D, K, B6, B9 and B12 were not considered in this analysis partly due to the absence of data in the Chinese Food Composition Table (for all five nutrients) and also because a sufficient status of vitamin D is difficult to achieve through diets alone and plant-based foods are not a relevant source of vitamin B12^26,27^. To assess the supply of these nutrients from plant-based foods in China, we conducted a supplementary analysis of coverage and source diversity for vitamins D, K, B6, B9 and B12 using data from the Japanese Food Composition Table^67^ (Supplementary Table 6 and Supplementary Notes).

### Quantitative networks linking crops and nutrients

A bipartite network of crops and nutrients per year at national or provincial levels was constructed in which the weight *W*_*tij*_ of the edge between crop *i* (30 crops or vegetable subgroups) and nutrient *j* (17 nutrients + energy) was equal to the ratio of the nutrient amount supplied by crop *i* and the intake need *μ*_*tj*_ for nutrient *j* in the whole population in year *t* (supply/demand ratio) (Supplementary Fig. 1):

1 $${W}_{{tij}}=\frac{{S}_{{ti}}\times {n}_{{ij}}}{{\mu }_{{tj}}}$$

in which *S*_*ti*_ is the quantity of crop *i* supplied for food consumption in year *t*, *n*_*ij*_ is the content of nutrient *j* in the edible portion of crop *i* and *μ*_*t*__*j*_ is the intake need for the whole population for nutrient *j* in year *t*. The supplied amount for food consumption for each crop (*S*_*ti*_) was calculated by using the domestic production plus international trade, corrected for stock changes and excluding non-food use (feed, seed, other uses), food loss and waste and inedible parts (Supplementary Fig. 1). To account for the impact of processing on nutrient content, we estimated nutrient supply from PFS considering particularities for each crop. We distinguished between refined grains and whole grains for rice and wheat, calculating their nutrient supplies separately (Supplementary Fig. 1 and Supplementary Notes). For soybean, groundnuts and sesame seed, we calculated separately the nutrient supply from soybeans, consumed as seeds and their derived vegetable oils. For sunflower seed, linseed and rapeseed, their supply amount for food consumption was converted to vegetable oil before calculating nutrient supply, to reflect their main use as vegetable oils for food consumption^56^. Finally, we summed up the nutrient supply from processed forms of each crop to estimate its total nutrient supply (Supplementary Fig. 1).

The quantity of each crop supplied for food consumption (*S*_*ti*_, g d^−1^) was calculated by multiplying the primary production amount of crop *i* in year *t* (*p*_*ti*_, tonnes yr^−1^), the loss ratios at the production (${l}_{i}^{1}$), postharvest handling (${l}_{i}^{2}$) and storage stages (${l}_{i}^{3}$). The net import of each crop (*f*_*ti*_) was added, and the non-food use was subtracted (*β*_*ti*_). Next, the loss and waste proportions (${l}_{i}^{h}$) for crop *i* at four stages *h* (processing, distribution, retail and consumption) were accounted for. We assumed that the loss and waste proportions did not change during 1997–2018. The annual supply amount (tonnes yr^−1^) was converted into the average daily amount in grams by multiplying it by $\frac{{10}^{6}}{365}$. Finally, the edible proportion of crop *i* (*α*_*i*_) was considered to estimate the quantity of each crop supplied for food consumption (equation (2)).

As described above, we disaggregated the total vegetable supply into seven subgroups *i* based on their shares (*θ*_*i*_) in consumption and calculated the quantity of each vegetable subgroup supplied for food consumption (equation (2a)). The total vegetable supply was calculated by considering the total production amount of vegetables *p*_*t*,veg_, loss ratios *l*_veg_^*h*^, net inflow *f*_*t*,veg_ and non-food use *β*_*t*,veg_. The seven subgroups of vegetables are listed above (‘Crops and nutrients considered’). However, even after disaggregating vegetables into subgroups, data limitations remain, and our estimates of diversity should be interpreted as lower bounds of actual diversity.

2 $${S}_{{ti}}=\left({p}_{{ti}}\times \mathop{\prod }\limits_{h=1}^{3}\left(1-{l}_{i}^{h}\right)+{f}_{{ti}}-{\beta }_{{ti}}\right)\times \mathop{\prod }\limits_{h=4}^{7}\left(1-{l}_{i}^{h}\right)\times \frac{{10}^{6}}{365}\times {{\rm{\alpha }}}_{i}$$

2a $$\begin{array}{lll}{S}_{ti} = \left({p}_{t,\mathrm{veg}}\times \mathop{\prod }\limits_{h=1}^{3}\left(1-{l}_{\mathrm{veg}}^{\,h}\right)+{f}_{t,\mathrm{veg}}-{\beta }_{t,\mathrm{veg}}\right)\\ \qquad \quad\times {\theta }_{i}\times \mathop{\prod }\limits_{h=4}^{7}\left(1-{l}_{i}^{h}\right)\times \frac{{10}^{6}}{365}\times {{\rm{\alpha }}}_{i}\end{array}$$

The nutrient needs for the whole population were calculated as a weighted average nutrient need in the population based on the nutrient need per capita within each age and sex group and the proportion of each stratum in the total population^68^, multiplied by the total population pop_*t*_ to get the demand at population level (equations (3a) and (3b)).

We used four different metrics to assess the requirement for 17 nutrients and dietary energy. For 16 nutrients (that is, all nutrients except potassium) we considered both the estimated average requirement (EAR) and the recommended nutrient intake (RNI). The EAR is the level of intake that meets the needs of 50% of all individuals in an age- and sex-specific stratum^29,30^. The RNI is the level of intake that meets the needs of 97.5% of individuals in each stratum^29,30^. Using the RNI as a reference value reduces the risk of insufficient supply for individuals but can overestimate what is needed for a population. This leads to two versions of equation (3) to estimate the required intake *μ*_*tj*_:

3a $${{\rm{\mu }}}_{{tj}}=\mathop{\sum }\limits_{k\in K}\left({\mathrm{ear}}_{{jkm}}\times {r}_{{kmt}}+{\mathrm{ear}}_{{jkf}}\times {r}_{{kft}}\right)\times {\mathrm{pop}}_{t}$$

3b $${{\rm{\mu }}}_{{tj}}=\mathop{\sum }\limits_{k\in K}\left({\mathrm{rni}}_{{jkm}}\times {r}_{{kmt}}+{\mathrm{rni}}_{{jkf}}\times {r}_{{kft}}\right)\times {\mathrm{pop}}_{t}$$

in which ear_*jkm*_, ear_*jkf*_ (rni_*jkm*_, rni_*jkf*_) were the intake need (EAR or RNI) of nutrient *j* in age group *k* for males and females, respectively. The proportions of males and females in age group *k* relative to the total population in year *t* were denoted as *r*_*kmt*_ and *r*_*kft*_, respectively. For potassium, only adequate intake (AI) was available and used^30^. For energy, the estimated energy requirement (EER) for a moderate level of physical activity was used^30^.

### Nutrient supply from animal-based food sources

We estimated nutrient supply from animal-based food sources using household-level consumption data from the 2011 China Health and Nutrition Survey^62^. We determined the average daily intake of dairy products, red meat, poultry, eggs and aquatic foods from 3-day 24-hour dietary recalls and linked it with the nutrient content of each food item to calculate the nutrient intake from animal-based food sources per capita (for more details, please refer to Chang et al.^65^).

### Nutrient coverage and source diversity

We calculated the coverage and source diversity for each nutrient. In Scenarios S1 and S2, the calculations included contributions from animal-based foods, which were aggregated and treated as a single source. The coverage NC_*tj*_ of nutrient *j* was calculated as the sum of supply/demand over all crop sources plus animal-based foods in year $t(\mathop{\sum }\limits_{i=1}^{I}{W}_{{tij}})$ where the demand (*μ*_*tj*_) was based on either EAR or RNI.

4 $${\mathrm{NC}}_{{tj}}=\mathop{\sum }\limits_{i=1}^{I}{W}_{{tij}}$$

We calculated source diversity as a measure of the number of equally abundant crop sources for providing nutrient *j*^28^. For nutrient *j* in year *t*, we calculated its Shannon entropy index *E*_*tj*_ as

5 $${E}_{{tj}}=-\mathop{\sum }\limits_{i=1}^{I}\left({e}_{{tij}}\mathrm{ln}\left({e}_{{tij}}\right)\right)$$

where *e*_*tij*_ equals $\frac{{W}_{{tij}}}{{\mathrm{NC}}_{{tj}}}$. Then, the source diversity SD_*tj*_ was computed as the exponential of the Shannon entropy^28^.

6 $${\mathrm{SD}}_{{tj}}=\exp \left({E}_{{tj}}\right)$$

Exponentiated Shannon entropy is the effective number of equally important sources supplying a nutrient^28^. It equals one when there is a single source and two when two sources supply equally. For three sources supplying 50, 25 and 25%, it equals 2.8. With four sources, supplying 50, 25, 24 and 1%, it equals 2.95. Source diversity accounts for both the number of sources and the evenness of their contributions to supply. The calculations of coverage and source diversity per nutrient and scenario analyses were developed and implemented using Python 3.7.4 in Spyder 3.3.6.

### Scenarios

We defined scenarios to analyse to what extent the plant-based food supply (PFS) can satisfy the nutrient needs in China (Table 1). Scenario S1 was the baseline scenario in which the coverage and source diversity per nutrient from PFS and animal-based foods were estimated. Scenarios S2–S3 explored how different strategies improved nutrient coverage and source diversity relative to Scenario S1.

Scenario S2a (reducing direct nutrient losses) assumed that food loss and waste for PFS were halved across all supply chain stages and 10% of refined rice and refined wheat flour would be replaced with whole grains alternatives.

Scenario S2b (reducing indirect nutrient losses by reducing red meat intake) assumed that red meat intake was reduced to the recommended level and it considered that crops originally used for feed would then potentially be available for direct food consumption (Supplementary Notes and Supplementary Table 7). This scenario assumption elucidates the amount of supply that could result from redirecting feed to food use, and it suggests opportunities for growing more nutritious food crops for direct consumption. This scenario inherited from Scenario S2a the reductions in food loss and waste, along with a 10% replacement of refined rice and wheat by whole-grain alternatives.

Scenario S3a (national self-sufficiency) assumed all domestically harvested food crops to be directly allocated to food consumption, without international trade. Thus, the domestic supply equalled domestic production minus seed and inedible parts. The assumption of reduced food loss and waste and an increase in whole grains were the same as in Scenarios S2a and S2b. For those crops that were partly used for food consumption and partly for vegetable oil processing (soybean, sesame seed and groundnuts) in Scenarios S1 and S2, the entire production was assumed to be supplied to consumers as soybeans and seeds rather than oil in Scenario S3a, given that nutrients mainly supplied by vegetable oils (for example, vitamin E) had sufficient coverage in the baseline.

Scenario S3b (provincial self-sufficiency) assessed the self-sufficiency and diversity of each province’s nutrient supply, without international or inter-provincial trade. The aim is to highlight the dependency of each province on imports from other provinces or from abroad. More details and the feasibility of scenarios from policy, technology and consumer behaviour perspectives are provided in Supplementary Information.

### Domestic crop production, international trade and crop use

Yearly production amounts of each crop in each province were obtained from the National Bureau of Statistics of China^55^ and China Rural Statistical Yearbook^69^. The crop import, export and stock changes were obtained from FAOSTAT^56^ Food Balance Sheets (FBS) (based on the old methodology from 1997 to 2009 and based on the new methodology from 2010 to 2018). The feed, seed and other uses (non-food) amounts per crop were also obtained from FBS (Supplementary Data)^56^. For four crops not listed in the FBS data (casaba, pears, watermelons, linseed), import and export data were obtained from FAOSTAT Trade data^56^.

To calculate the supply of each crop for food consumption, we matched crop production data from the National Bureau of Statistics with FAOSTAT data on imports, exports, stock changes and crop uses (Supplementary Data). The amount of vegetable oil processed from soybean, groundnuts, sunflower seed, rapeseed, sesame seed and linseed was calculated by considering the oil extraction rate of each crop^10,70^ and the shelling rate of groundnuts (0.7)^70^.

### Food loss and waste

The loss proportions and the waste proportions for each crop were obtained from Xue et al.^6^. The loss proportions of vegetable oil (for example, linseed oil, groundnut oil and so on) were obtained from Wang et al.^11^.

### Edible proportion and nutrient content of foods

To estimate the nutrient supply from plant-based foods, supply data for each crop were matched with corresponding nutrient content values (matching procedure described in Supplementary Data 3). Nutrient content was based on minimally processed, uncooked plant-based foods. Edible proportions and nutrient contents were estimated as follows.

For vegetables, we disaggregated total supply into seven subgroups (specific groups see above ‘Crops and nutrients considered’) by using their consumption shares^62^. To estimate subgroup-specific edible proportions and nutrient content, we used equally weighted averages of fresh food items within each subgroup, excluding dried and canned products.

For single crop species (for example, rice, wheat and bananas), we used the representative values (that is, the median after removing outliers) or a unique value for their raw agricultural food products obtained from the Chinese Food Composition Table^58^. For crops with multiple sub-products in the food composition tables (for example, maize, including white and yellow maize flour), nutrient content and edible proportions were estimated using consumption-weighted averages based on the China Health and Nutrition Survey^62^. Similarly, for categories comprising multiple crop species (for example, other fruits and other beans), consumption-weighted averages were used. For the ‘other cereals’ category, due to discrepancies in consumption shares between the China Health and Nutrition Survey^62^ and a previous study based on the Chinese Nutrition and Health Surveillance^71^, an equal-weighted average was used to estimate nutrient content and edible proportion.

We obtained the selenium and niacin content for brown rice^72,73^ and the vitamin E content for linseed oil from previous studies^74^, due to the absence of data in the Chinese Food Composition Table^58^. Niacin in brown rice was estimated as the mid-point of the range reported in Zhou and Zhang^73^.

### Dietary nutrient needs

To estimate the nutrient intake needs of the Chinese population, we calculated the per capita intake needs for all age and sex groups, weighted by their proportion in the total population, and then multiplied by the entire population. Higher needs for pregnant and lactating women were not included in the age- and sex-weighted averages in our scenario analyses but were assessed in a supplementary analysis (Supplementary Table 8), estimating the percentage increase in total dietary intake needs when these higher requirements were considered.

The per capita intake needs were based on EAR, RNI and AI (adequate intake, only for potassium) and EER (estimated energy requirement, for a moderate level of physical activity for dietary energy), obtained from the Chinese dietary reference intakes (DRIs)^30^ and the dietary guidelines for Chinese residents (2022)^75^. Due to the lack of RNI values for vitamin E, carbohydrates and dietary fibre in Chinese recommendations, their recommended intakes were obtained from the US National Academies of Sciences, Engineering and Medicine^76,77^.

National age and sex group in the overall population^78^, annual provincial population data (1997–2018)^78,79^ and the province-specific sex and age group proportions in 2018^78^ were obtained from the National Bureau of Statistics of China.

### Uncertainty assessment

**Extended Data Fig. 7:**
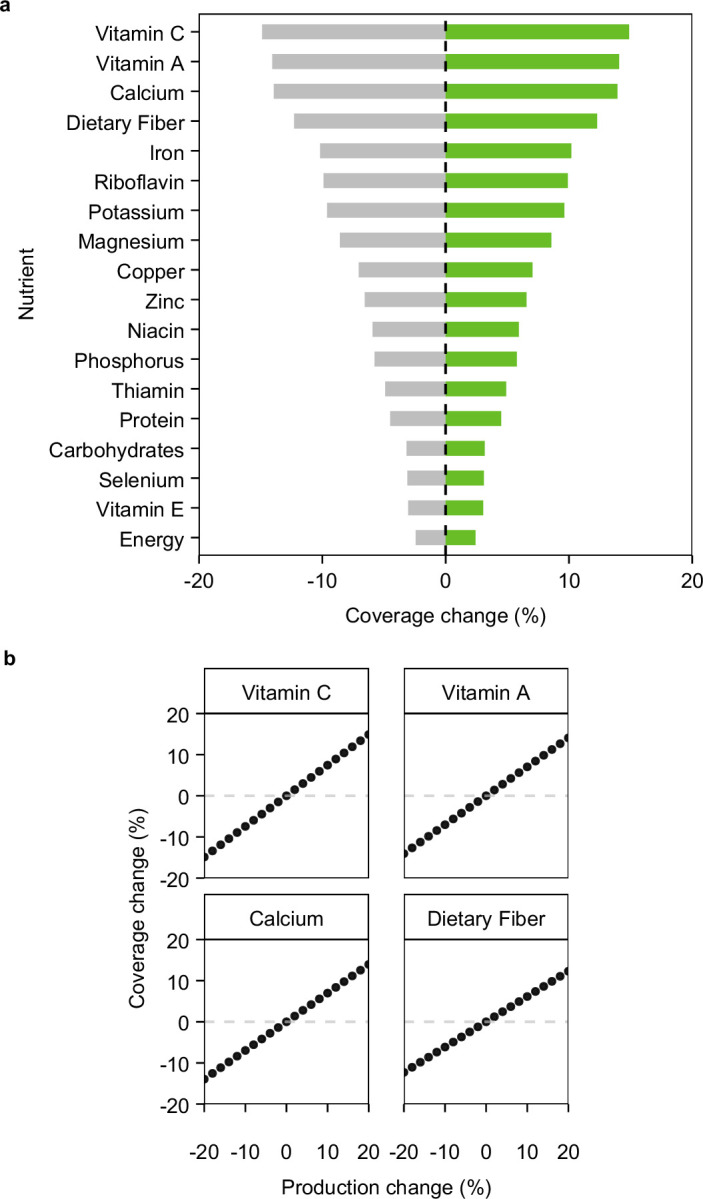
Effect of production volume of vegetables on the coverage for seventeen nutrients and dietary energy in baseline Scenario S1. Panel **a** shows the change in coverage relative to the estimated average requirement if the production amount of total vegetables is increased (green bars) or decreased (grey bars) by 20%. Panel **b** shows an example of the linear relationships for four nutrients (vitamins C and A, calcium and dietary fibre) when the vegetable production amount is changed in 2% increments from the current value. The total vegetable supply was divided into seven subgroups in this sensitivity analysis by using their consumption shares (as in the main analysis). Source data

We used several approaches to cross-check data and assess uncertainties. First, integrating provincial crop production data from China’s National Bureau of Statistics (NBS) with national trade and crop use data from FAOSTAT may introduce inconsistencies. We compared the national production amounts for crops from the two data sources (Supplementary Fig. 2) and evaluated the impact of discrepancies between them. We evaluated the impact by adjusting the crop production amount with the largest discrepancies (vegetables) by ±20% in 2% increments and then reassessed coverage per nutrient under Scenario S1 (Extended Data Fig. 7).

A second source of uncertainty is data aggregation. Limited data availability on production resulted in grouping some crops and vegetable subgroups (for example, stem, leafy and flower vegetables, other fruits), while there is variation in nutrient content within these groups. We used Monte Carlo simulations to assess uncertainties from data aggregation (Extended Data Fig. 6). For details, see the ‘Discussion’ section (main text) and Supplementary Notes section (Supplementary Information file).

### Reporting summary

Further information on research design is available in the Nature Portfolio Reporting Summary linked to this article.

## Supplementary information

Supplementary Information Supplementary Figs. 1–3, Tables 1–8, discussion and notes.

Reporting Summary

Supplementary Data Supplementary datasets supporting the results of the study.

## Source data

Source Data Fig. 1 Source data for Fig. 1.

Source Data Fig. 2 Source data for Fig. 2.

Source Data Fig. 3 Source data for Fig. 3.

Source Data Fig. 4 Source data for Fig. 4.

Source Data Fig. 5 Source data for Fig. 5.

Source Data Extended Data Fig. 1 Source data for Extended Data Fig. 1.

Source Data Extended Data Fig. 2 Source data for Extended Data Fig. 2.

Source Data Extended Data Fig. 3 Source data for Extended Data Fig. 3

Source Data Extended Data Fig. 4 Source data for Extended Data Fig. 4.

Source Data Extended Data Fig. 5 Source data for Extended Data Fig. 5.

Source Data Extended Data Fig. 6 Source data for Extended Data Fig. 6.

Source Data Extended Data Fig. 7 Source data for Extended Data Fig. 7.

## Figures and Tables

**Extended Data Table 1:**
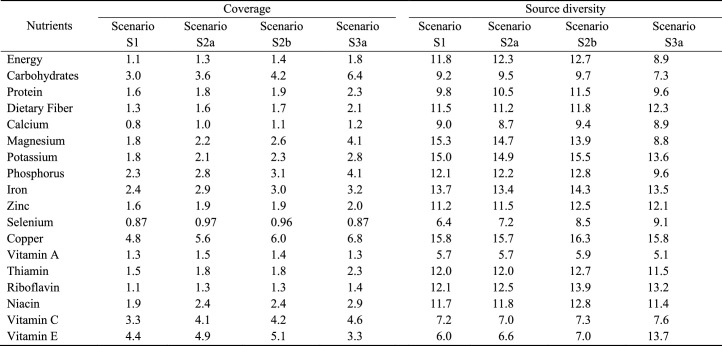
Coverage and source diversity per nutrient at the national level in Scenarios S1, S2a, S2b, and S3a

Coverage (supply/demand ratio) is expressed relative to the estimated energy requirement for energy, relative to the adequate intake for potassium, and relative to the estimated average requirement (EAR) for the other nutrients. Scenario S1 represents the baseline. In Scenario S2a, we reduced direct nutrient losses by reducing food loss and waste in the supply chain and at consumers along with a small increase (10%) in use of whole grains for rice and wheat. In Scenario S2b, we reduced indirect nutrient losses by decreasing red meat intake, which frees up crops used for animal nutrition as a potential source for human consumption. Scenario S3a aimed to analyse which level of self-sufficiency could be reached when all domestically produced food crops would be used for direct human consumption and international trade was absent.

## Data Availability

The datasets supporting the findings are available from the cited references, the Supplementary Information and Supplementary Data 1. Supplementary Data 1 contains the list of sources leading to the original data sources. Source data are provided with this paper. These data are also available via Zenodo at 10.5281/zenodo.19036893 (ref. ^80^).

## Code availability

The code used for the scenario analyses in the study is available via Zenodo at 10.5281/zenodo.19036893 (ref. ^80^).
