# Supplementary material for: Nutrient coverage of China’s plant-based food supply can be improved with food system adjustments
Source: Nat Food. 2026 May 1;7(5):452–63. doi: 10.1038/s43016-026-01349-6 (PMC13212153; doi:10.1038/s43016-026-01349-6)
Supplement: Supplementary file 1 — Supplementary Figs. 1–3, Tables 1–8, discussion and notes. [file 43016_2026_1349_MOESM1_ESM.pdf]

nature food

Supplementary information https://doi.org/10.1038/s43016-026-01349-6

Nutrient coverage of China's plant-based food supply can be improved with food system adjustments

In the format provided by the authors and unedited

Supplementary Information

Contents

Supplementary Figures 1-3

Supplementary Tables 1-8

Supplementary Discussion

Further discussion on scenarios . . . .

Supplementary Notes

Assessing indirect nutrient loss reduction through reduced red meat intake (Scenario S2b) . . . .

Nutrient supply from plant-based foods for vitamins D, K, B6, B9, and B12

Rice and wheat supply for food consumption . . . .

Comparison of the effects of individual measures to use nutrients more efficiently (Scenario S2) . . . .

Impact of uncertainty in baseline coverage and source diversity due to low resolution of supply data . . . .

Supplementary Figures 1-3

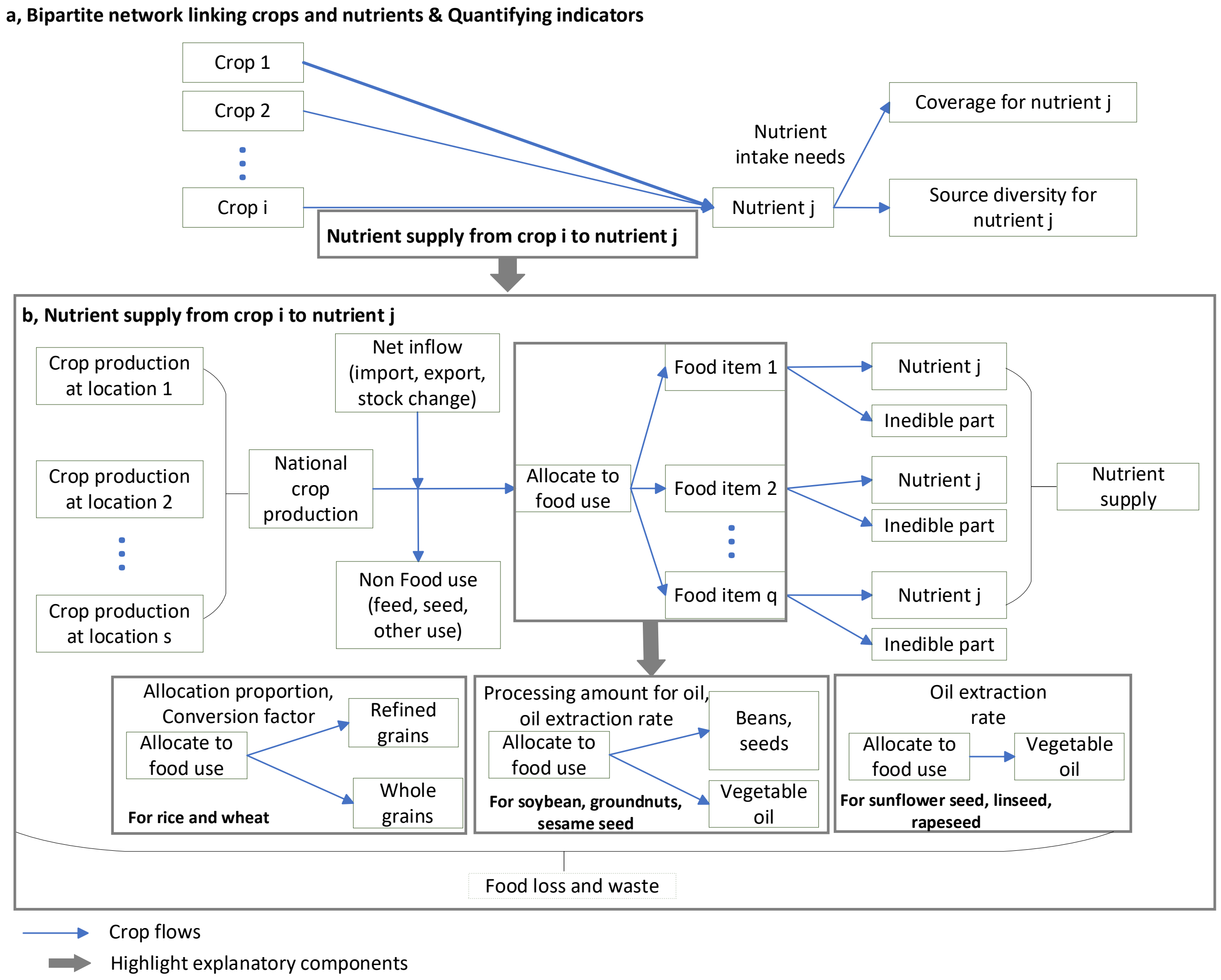

Supplementary Fig. 1 | Framework for constructing the bipartite networks of crops and nutrients and estimating the nutrient supply from all crop sources. Panel A illustrates how coverage for a nutrient j and the diversity of supply for this nutrient are quantified on the basis of supply from different crops in relation to nutrient intake needs. Panel B illustrates the procedure. First, the domestic supply of each crop allocated to food use was calculated. This takes into account the national crop production (aggregating production from all provinces), adding net inflow (imports, exports, and stock changes), and subtracting non-food uses (e.g., industrial purposes) and food loss. Then, based on the food-use supply of each crop, the nutrient supply was further calculated. This requires linking each crop to its associated plant-based foods and using nutrient content data to quantify the nutrient supply from each crop. Given that food processing may change nutrient content, we estimated nutrient supply for certain crops by summing across the nutrient supply from their processed products: for rice and wheat, refined grains and whole grains were calculated separately and then added up; for soybean, groundnuts, and sesame seed, the nutrient supplies from beans, seeds, and their derived vegetable oils were calculated separately and then added up. Finally, inedible parts and consumer-stage food waste were excluded.

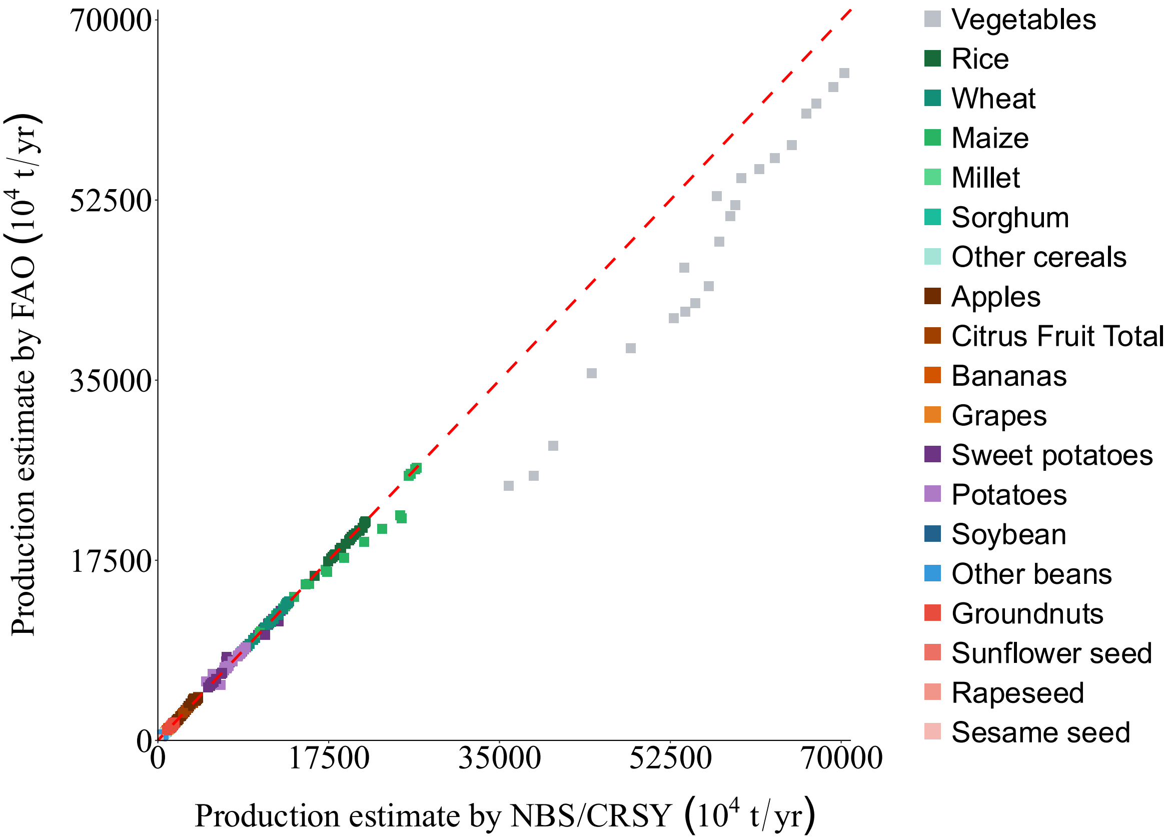

Supplementary Fig. 2 | Comparison of production amount from 1997 to 2018 according to FAOSTAT Food Balance Sheets[1] and according to the National Bureau of Statistics (NBS) of China[2] and China Rural Statistical Yearbook[3]; CRSY). The red dashed line is the 1:1 line. The crop production data at the subnational level (i.e., provincial level) are only available from NBS, while national-level data on crop imports, exports, and crop use are only provided by FAOSTAT. Thus, to calculate the domestic supply per crop allocated to food use and support provincial-level analysis of nutrient supply, we integrated data from both sources. Data from CRSY were used to supplement missing values in the NBS dataset where necessary. To evaluate the differences between the datasets, we compared annual crop production from the two datasets. However, for five crops/crop groups analyzed in this study (pears, watermelons, casaba, other fruits, and linseed), FAOSTAT does not provide national-level production data. Therefore, the comparison was limited to the 19 crops/crop groups with available data. The production comparison in this figure does not have the subdivision into seven subgroups of vegetables. In the scenario analyses presented in the main text, total vegetable supply was disaggregated into seven subgroups based on their consumption shares to better capture nutrient content and reflect the diversity of vegetables. The results showed that most crop production amounts had consistency, whereas vegetable production reported by the NBS was higher than that reported by FAOSTAT.

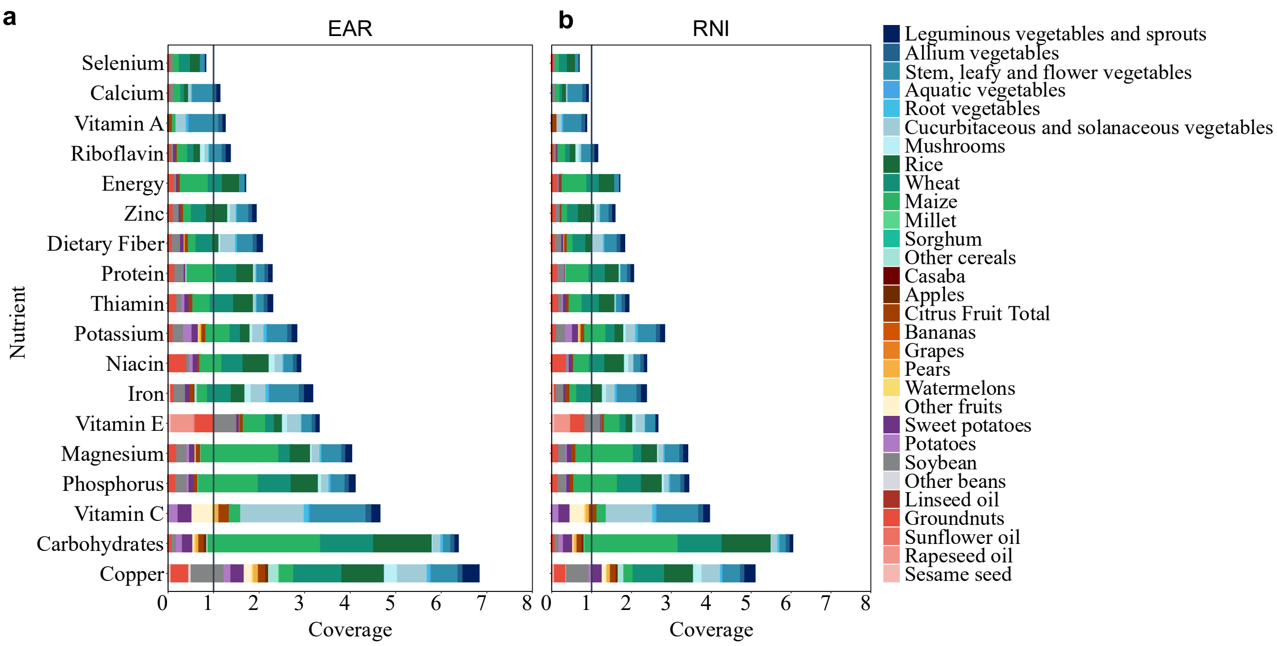

Supplementary Fig. 3 | Coverage of 17 nutrients and dietary energy by 30 crops in Scenario S3a, with coverage broken down by plant-based food sources. Scenario S3a (Self-sufficiency) aims to analyze which level of self-sufficiency could be reached when all domestically produced food crops would be used for direct human consumption and international trade was absent but with inter-provincial trade. Coverage from plant-based food sources was based on EAR (estimated average requirement) in panel A, and based on RNI (recommended nutrient intake) in panel B. For potassium, coverage was in both panels expressed relative to adequate intake (AI) and for energy, coverage was in both panels expressed relative to estimated energy requirement (EER). Coverage from oil crops was separated in the part provided by the oil and directly by the seeds, as appropriate.

Supplementary Tables 1-8

Supplementary Table 1 | The percentage contribution from plant-based and animal-based food sources to the total coverage for each nutrient in Scenarios S1, S2a, and S2b. Scenario S1 represents the current situation in 2018. Scenario S2a assumes reducing food loss and waste in the supply chain and including a small increase (10%) in whole grains supply. Scenario S2b assumes lower indirect nutrient losses by decreasing red meat intake, which frees up crops used for animal feed as a potential source for human consumption. The percentage contribution does not depend on whether EAR (estimated average requirement) or RNI (recommended nutrient intake) is used as a reference intake. For potassium and energy, only adequate intake (AI) and estimated energy requirement (EER) are available and used.

Nutrients	Scenario S1 Baseline		Scenario S2a		Scenario S2b	
	Plant-based	Animal-based	Plant-based	Animal-based	Plant-based	Animal-based
Energy	87	13	89	11	93	7
Carbohydrates	99	1	99	1	99	1
Protein	74	26	77	23	83	17
Dietary Fiber	100	0	100	0	100	0
Calcium	87	13	89	11	90	10
Magnesium	94	6	95	5	97	3
Potassium	90	10	92	8	95	5
Phosphorus	83	17	86	14	90	10
Iron	88	12	90	10	93	7
Zinc	80	20	83	17	88	12
Selenium	56	44	60	40	67	33
Copper	93	7	94	6	96	4
Vitamin A	73	27	77	23	82	18
Thiamin	86	14	88	12	94	6
Riboflavin	76	24	79	21	85	15
Niacin	81	19	84	16	90	10
Vitamin C	100	0	100	0	100	0
Vitamin E	98	2	98	2	98	2
Average	86	14	88	12	91	9

Supplementary Table 2 | Percentage of different sections of the Chinese population with inadequate micronutrient intake from their actual diets according to three published studies. Each study addressed intake in a different section of the population. Adequacy was judged by comparing actual intake with estimated average requirement (EAR) or adequate intake (AI). NR stands for not reported. Vitamin A was reported as retinol activity equivalents (RAE) or retinol equivalent.

		2010-2013[4] 2 years and older	2015-2017[5] 18 years and older	2015-2017[6] 2 years and older
Vitamin A (µgRAE) or retinol equivalent (µg)*	<EAR	90.6	85.7	75.9 (retinol)
Thiamine (Vitamin B1)	<EAR	77.0	86.7	83.5
Riboflavin (Vitamin B2)	<EAR	89.0	96.0	88.9
Niacin (Vitamin B3)	<EAR	14.8	38.7	NR
Vitamin C	<EAR	67.5	63.7	68.9
Vitamin E	<AI	NR	21.2	NR
Calcium	<EAR	96.3	97.6	97.2
Iron	<EAR	13.5	19.4	12.0
Zinc	<EAR	35.4	51.9	36.3
Phosphorus	<EAR	15.2	23.8	NR
Magnesium	<EAR	60.6	70.5	NR
Manganese	<EAR	NR	77.1	NR
Copper	<EAR	NR	2.6	NR
Selenium	<EAR	70.8	81.7	NR
Potassium	<AI	NR	85.5	NR

[4] Zhao L, He Y, Yang Y, Yu D, Wang Z. *China nutrition and health surveillance (2010-2013): the dietary and nutritional status of Chinese population (in Chinese)*. People's Medical Publishing House (2018).

[5] Huang K*, et al.* Usual Intake of Micronutrients and Prevalence of Inadequate Intake among Chinese Adults: Data from CNHS 2015-2017. *Nutrients* **14**, (2022).

[6] Yu D*, et al.* Status of energy and primary nutrients intake among Chinese population in 2015-2017 (in Chinese). *Food and Nutrition in China* **27**, 5-10 (2021).

[*] Vitamin A was reported as retinol activity equivalents (RAE) in Zhao et al. (2018) and Huang et al. (2022), and as retinol equivalent in Yu et al. (2021).

Supplementary Table 3 | Percentage of individuals with micronutrient deficiency or insufficiency in different Chinese population strata, based on biomarker evidence from published studies. Biomarker evidence, deficiency and insufficiency cutoff values are provided in the Supplementary Data file.

Nutrients	Outcome indicator	Age group	Percentage of deficiency or insufficiency	Reported year	Geographic representation
**Calcium**	deficiency[7]	Children	34%	2004-2005	Jintan, Jiangsu province
**Selenium**	deficiency[8]	6-11-year-old	9.4% (very low selenium)	2012	National, rural areas
	deficiency[8]	6-11-year-old	25.6% (low selenium)	2012	National, rural areas
**Vitamin A**	deficiency[9]	0-17-year-old	0%~27%	2010-2019	Several provinces' value based on literature review of papers (2010-2022)
	insufficiency[9]	0-17-year-old	15%~58%	2010-2019	Several provinces' value based on literature review of papers (2010-2022)
	deficiency[10]	6-17-year-old	1%	2016-2017	National
	marginal deficiency[10]	6-17-year-old	15%	2016-2017	National
	deficiency[10]	above 18-year-old	1%	2016-2017	National
	marginal deficiency[10]	above 18-year-old	4%	2016-2017	National
	deficiency[11]	Age-standardized	2%	2019	National, secondary trend analysis based on the Global Burden of Disease 2019
**Zinc**	deficiency[12]	6-11-year-old	10%	2012	National, rural areas
	deficiency[8]	6-17-year-old	11%	2016-2017	National
	deficiency[8]	above 18-year-old	6%	2015	National
**Magnesium**	deficiency[11]	Children	0.6%	2004-2005	Jintan, Jiangsu province
**Iron**	deficiency[7]	0-18-year-old	7.3% ~ 62%	2010-2018	National value based on literature review of papers (2010-2022)
	Anemia (mild)[10]	0-5-year-old (exclude 0-6 months)	13.7%	2016-2017	National
	Anemia[10]	6-11-year-old	4.4%	2016-2017	National
	Anemia[10]	11-17-year-old	6.6%	2016-2017	National
	Anemia[10]	above 18-year-old	8.3%	2015	National
	Dietary iron deficiency[11]	Age-standardized	0.05%	2019	National, secondary trend analysis based on the Global Burden of Disease 2019
**Copper**	Copper deficiency[13]	Children	0.7%	2004-2005	Jintan, Jiangsu province
**Iodine**	deficiency[13]	8-10-year-old	2.2%	2011	National
	deficiency[11]	Age-standardized	1.4%	2019	National, secondary trend analysis based on the Global Burden of Disease 2019
**Vitamin D**	deficiency[9]	0-18-year-old	20%-30%	2011-2017	Several provinces' value based on literature review of papers (2010-2022)
	deficiency[10]	6-17-year-old	19%	2016-2017	National
	insufficient[10]	6-17-year-old	43%	2016-2017	National
	deficiency[10]	above 18-year-old	21%	2015	National
	insufficient[10]	above 18-year-old	39%	2015	National
	deficiency[14]	above 18-year-old	21%	1995-2019	National value based on meta analysis of papers (2001-2021)
	inadequacy[14]	above 18-year-old	63%	1995-2019	National value based on meta analysis of papers (2001-2021)
	deficiency[14]	Children and adolescents	23%	1995-2019	National value based on meta analysis of papers between (2001-2021)
	inadequacy[14]	Children and adolescents	47%	1995-2019	National value based on meta analysis of papers (2001-2021)
**Vitamin B12**	deficiency[15]	6-23-months of age	0%	2018	Huzhu county, Qinghai province
	deficiency[16]	12-23-months of age	20%	2010	Three counties (Wuding, Zhengan, and Zhenan)

Supplementary Table 4 | National average nutrient production per hectare of crops in China in 2018. Nutrient production per hectare of each crop was calculated based on the national average yield per hectare, nutrient content, and conversion factors from harvested grains to processed grains. For refined rice and brown rice, the yield per hectare is the same, but their nutrient production differs due to differences in nutrient content and the conversion factors from harvested rice to refined rice and brown rice. The same reason applies to the difference in nutrient production between refined wheat flour and whole wheat flour.

Nutrients	Unit	Crops							
		Millet	Sorghum	Mung bean	Adzuki bean	Brown rice	Refined rice	Whole wheat	Refined wheat
Calcium	kg/ha	1.2	0.9	1.5	1.5	0.6	0.3	1.4	0.8
Riboflavin	g/ha	3.0	4.2	2.0	2.2	2.2	1.5	4.1	1.8
Iron	g/ha	148.7	262.2	120.8	149.0	99.8	40.1	208.6	42.5
Dietary Fiber	kg/ha	45.9	179.0	119.0	155.0	116.4	21.9	441.7	24.3
Energy	10^6 kcal/ha	10.9	15.0	5.9	6.2	19.3	12.6	13.8	10.9

Supplementary Table 5 | Effect of food preparation on the coverage of 17 nutrients and dietary energy in the baseline Scenario S1. To understand the impact of cooking on nutrient supply, we tested both the worst-case (e.g., the cooking method that may lead to the highest loss of nutrients) and the common method (stir-frying). This was done for vegetables only. In the case of preparation of vegetables by boiling, the assumption was made that the cooking water was discarded and not used for consumption. The boiling method results in the highest nutrient losses among all cooking methods and thus can represent a worst-case. The data on nutrient changes of vegetable subgroups after specific cooking methods were obtained from ref.[17]. The data on nutrient change after boiling and stir-frying were not available for three subgroups: allium vegetables, aquatic vegetables, leguminous vegetables and sprouts. For these, we used the average values of the nutrient loss ratio after boiling or stir-frying of the other four vegetable subgroups (root vegetables; cucurbitaceous and solanaceous vegetables; stem, leafy, and flower vegetables; mushrooms). Nutrient supply calculations in our scenario analyses did not take into account the effects of food preparation on nutrient content in vegetables.

Nutrients	Coverage EAR					Coverage RNI	
	Fresh	Boiling		Stir-frying		Boiling	Stir-frying
		After boiling	Percentage change (%)	After stir-frying	Percentage change (%)	After boiling	After stir-frying
Energy	1.1	1.1	0.0	1.1	0.0	1.1	1.1
Carbohydrates	3.0	3.0	-1.4	3.0	0.0	2.8	2.9
Protein	1.6	1.5	-2.0	1.5	-1.0	1.4	1.4
Dietary Fiber	1.3	1.2	-3.6	1.3	0.0	1.1	1.1
Calcium	0.8	0.8	-3.1	0.8	0.0	0.7	0.7
Magnesium	1.8	1.5	-15.5	1.8	0.0	1.3	1.5
Potassium	1.8	1.4	-21.9	1.7	-3.5	1.4	1.7
Phosphorus	2.3	2.3	-2.6	2.3	0.0	1.9	1.9
Iron	2.4	2.1	-11.5	2.4	0.0	1.6	1.8
Zinc	1.6	1.5	-7.3	1.6	0.0	1.2	1.3
Selenium	0.9	0.9	-1.4	0.9	0.0	0.7	0.7
Copper	4.8	4.7	-1.4	4.8	0.0	3.5	3.6
Vitamin A	1.3	1.2	-6.1	1.2	-6.1	0.8	0.8
Thiamin	1.5	1.4	-6.0	1.5	-0.7	1.2	1.2
Riboflavin	1.1	0.9	-14.8	1.1	-0.4	0.8	0.9
Niacin	1.9	1.8	-9.3	1.9	-1.4	1.4	1.6
Vitamin C	3.3	2.3	-31	2.9	-13.2	1.9	2.4
Vitamin E	4.4	4.4	0.0	4.4	0.0	3.6	3.6

Supplementary Table 6 | Coverage and source diversity for vitamins D, K, B6, B9, and B12 from plant-based foods. The dash for the source diversity of vitamin B12 indicate that this indicator is not applicable because vitamin B12 is essentially from animal-based foods. These five nutrients were not included in our main analysis because of the absence of nutrient content of these nutrients in Chinese food composition tables and because it is widely acknowledged that sufficient vitamin D intake is difficult to achieve through diets alone and vitamin B12 is essentially only from animal-based foods. To obtain estimates of coverage and source diversity for these five nutrients, we calculated the supply from all plant-based foods for food use in China in 2018 using nutrient content data for these nutrients from the Japanese Food Composition Table[18]. Mushrooms are treated as a vegetable subgroup although they are biologically fungi. Mushrooms, which have been suggested as potential source of vitamin D, do not provide meaningful vitamin D amounts unless they are UV-exposed[19]. Even then, they provide vitamin D2, which is less effective than vitamin D3 at increasing vitamin D levels in the human body and may even reduce the effectiveness of vitamin D3[20]. Nutrient intake needs, including the estimated average requirement (EAR), recommended nutrient intake (RNI), and adequate intake (AI) for vitamin K were obtained from the Chinese Dietary Reference Intakes[21].

Nutrients	Coverage EAR	Coverage RNI	Source diversity
Vitamin D (from mushrooms)	0.04	0.03	1
Vitamin K	11	11	3.4
Vitamin B9	2.6	2.1	8.3
Vitamin B6	2.2	1.8	12.1
Vitamin B12	0	0	-

Supplementary Table 7 | Production output of crops normally used as animal feed (and used as such in Scenario S1) that is released for direct human consumption in Scenario S2b. Scenario S2b (Reduce indirect nutrient losses by reducing red meat intake) explores changes in nutrient supply when repurposing crops originally used for feed for potential direct consumption as food, assuming that red meat intake was reduced to the recommended level and the associated production of feed from arable crop production (mainly grain maize, tubers, and soybean) could be redirected towards human consumption. In this scenario, we assumed that red meat intake could be reduced to align with the total meat consumption levels recommended by the Chinese Dietary Guidelines[22]. Then, the amount of food crops previously used as animal feed that could be repurposed for direct human consumption can be calculated (for more details refer to Supplementary Notes below). Data for the original feed use amount was obtained from the FAOSTAT Food balance sheet[1] and feed use proportion for different livestock sectors was obtained from Fang et al. (2023)[23]. The released soybean and sesame seed were assumed in Scenario S2b to be used directly as beans and seeds rather than for producing oil.

Crop	Released feed (10^3 tonnes)
Maize	50159
Vegetables	7659
Sweet potatoes	6021
Wheat	4005
Potatoes	3468
Soybean	2553
Rapeseed	1187
Other beans	1022
Other cereals	776
Rice	757
Sorghum	622
Sunflower seed	562
Millet	289
Sesame seed	22

Supplementary Table 8 | Percentage increase (%) in dietary nutrient needs of the whole population considering the higher needs of pregnant and lactating women compared to the estimation of age- and sex-weighted average dietary needs in our main analysis. EAR is the estimated average requirement; RNI is the recommended nutrient intake (RNI); Only the adequate intake (AI) for potassium is available and used; for energy, only the estimated energy requirement (EER) for a moderate level of physical activity is used. We obtained the higher nutrient needs for pregnant and lactating women from the Chinese DRIs Handbook (2013), estimating nine months of pregnancy and six months of lactation. These were multiplied by the proportion of this group in the total population (represented by the birth rate in 2018, which is 1.09%[2]) to calculate the higher daily nutrient needs per capita. Then, we calculated the percentage increase in the nutrient needs relative to the needs estimated based on the age- and sex-weighted average used in our analysis. Higher needs for pregnant and lactating women were not included in the age- and sex-weighted averages in our scenario analyses.

Nutrients	EAR	RNI	EER	AI
Energy			0.2	
Protein	0.4	0.5		
Dietary Fiber		0.2		
Vitamin A	0.5	0.5		
Thiamin	0.2	0.2		
Riboflavin	0.2	0.2		
Vitamin C	0.3	0.4		
Calcium	0.2	0.2		
Iron	0.4	0.4		
Zinc	0.4	0.4		
Carbohydrates	0.2	0.6		
Niacin	0.1	0.1		
Phosphorus	0.0	0.0		
Magnesium	0.1	0.1		
Selenium	0.2	0.2		
Copper	0.6	0.5		
Vitamin E	0.2	0.2		
Potassium				0.1

Supplementary Discussion

Further discussion on scenarios

In addition to a baseline Scenario (S1) for the year 2018, this study considered two change scenarios (Scenarios S2 and S3). Here, we discuss the feasibility of Scenarios S2 and S3 from policy, technology, and consumer behavior perspectives.

Scenario S2 (Reducing nutrient losses) focuses on improving nutrient efficiency from plant-based food supply (PFS) by addressing both direct and indirect nutrient losses. Scenario S2a targets direct losses by halving food loss and waste and using more whole grains. Scenario S2b builds on Scenario S2a by additionally assuming a reduction in red meat intake and repurposing crops from animal feed to direct human consumption. Reducing food loss and waste is part of the global sustainable development goals and has received worldwide attention. In China, regulations such as the 'Anti-food Waste Law of the People's Republic of China' have been introduced to curb food waste behavior[24]. A policy on 'Scientific Grain Storage Projects' was introduced to provide subsidies for farmers to buy advanced storage facilities, including metal silos and sealed bags, to reduce food loss in farmer household storage[25]. Such policies reflect ongoing efforts and attainable success in halving food loss and waste.

Second, refined grains are less nutritious than whole grain alternatives, and high consumption of refined grains is associated with increased risks of mortality and nutrition-related chronic diseases[26]. Whole grains are currently emphasized for their advantages in promoting healthy diets and are highlighted in food processing technology. The 'Development Plan for the Grain Processing Industry (2011-2020)' in China emphasizes the need for technological breakthroughs in whole-grain foods based on ingredients such as brown rice and whole wheat[27]. Whole grains are part of China's Dietary Pagoda, which can contribute to healthy diets[28]. These advancements underscore the practical significance of understanding the effects of whole grain substitution on the sufficiency and diversity of available nutrients.

Scenario S2b (Reducing indirect nutrient losses by reducing red meat intake) explores the change in nutrient coverage and source diversity from repurposing crops currently used as feed to food production. This scenario assumes that the red meat intake (ruminant meat and pork) can be reduced to align total meat consumption with balanced dietary guidelines in China[22,28]. Despite studies confirming that increased red meat consumption is linked to a higher risk of nutrition-related chronic diseases, such as type 2 diabetes[29], and worsening environmental impacts[30,31], the proportion of the population having excessive red meat intake in China (≥100 g capita^{-1} day^{-1}) rose from 33% in 2013 to 42% in 2018[32]. Simultaneously the Chinese dietary guidelines recommend meat intake of maximally 25 grams per 1000 kcal[22], so around 55 g capita^{-1} day^{-1}. Meat consumption is driven by various factors such as economic growth and urbanization, yet the willingness to consume meat can be influenced by information campaigns[33,34]. A study in New Zealand found that providing information about meat's impact on the climate can decrease the willingness to consume meat[35]. This indicates that it is possible to reduce red meat intake through informational interventions and nutrition education.

Scenario S3 (Self-sufficiency) examines the achievable level of self-sufficiency when all domestically harvested food crops are used exclusively for direct human consumption, with no international trade (Scenario S3a) and also without inter-provincial trade (Scenario S3b). In Scenario S3b, the provincial plant-based food supply was calculated as provincial production minus the amounts allocated to seed use and inedible parts. The seed use per crop in each province was determined based on the national seed use for each crop in 2018 and distributed to each province according to its share of the crop's total national production. Our results indicate that if domestically harvested food crops are not allocated to other uses (e.g., feed or industrial use), the supplied amounts without international trade for food consumption can greatly contribute to nutrient coverage. This assumption is extreme and will not happen in this form. Because a large-scale direct repurposing of maize for direct consumption by humans is only theoretically possible and cannot happen in the real world as the quantity of feed and non-food uses is too large to be consumed by humans (Supplementary Fig. 3). However, the extreme

scenario serves to make the point that once the allocation of harvested food crops to different uses can be changed, opportunities will emerge. The arable land could be used to grow more nutrient-dense crops or simultaneously alleviate pressure on land and other resources[36]. This scenario highlights the potential for changes in the allocation of crop uses, revealing the possibility of adopting more balanced diets and a food system that is more nutritious, sustainable, and robust.

Supplementary Notes

Assessing indirect nutrient loss reduction through reduced red meat intake (Scenario S2b)

Scenario S2b explores changes in nutrient supply under conditions of reduced food loss and waste, an increased supply of whole grains, and reduced red meat intake to align with the total meat consumption levels recommended by the Chinese Dietary Guidelines[22]. We calculated the amount of food crops used as animal feed in Scenario S1 that could be repurposed for direct human consumption due to the reduction in red meat intake. The procedure was as follows. According to the China Health and Nutrition Survey (CHNS 2011), the average daily energy intake was 2194 kcal per capita. The average daily meat consumption was 88 grams per capita, including 74 grams of red meat (64.2 grams of pork, 7.5 grams of beef, 1.8 grams of lamb, 0.4 grams of other red meats[37]), and 14 grams of poultry. However, the Chinese national dietary guidelines recommended meat intake no more than 25 grams per 1000 kcal[22]. The current average daily per capita meat consumption exceeded this recommendation by 33 grams. Thus, we explored the impact of reducing red meat intake (pork, beef, lamb, other red meats) with 33 grams per capita per day, which is a reduction of 45% (33/(64.2+7.5+1.8)). The impact of reducing other red meat was not included, given that the current intake was only 0.4 grams per capita per day. Then, 45% of the feed amounts from crops initially used for meat production were reassigned to food consumption use, in which the released soybean and sesame were assumed to be used as beans and seeds rather than producing oil (Supplementary Table 7). The proportion of feed amounts from food crops used for different livestock sectors (e.g., pigs, beef, lamb, etc.) was obtained from ref.[23].

Nutrient supply from plant-based foods for vitamins D, K, B6, B9, and B12

To have an overview of more nutrients not included in our analysis due to the absence of data, we conducted a supplementary analysis on vitamins D, K, B6, B9, and B12, using the Japanese food composition table[18].

We assessed the coverage and source diversity of vitamins D, K, B6, B9 (folate), and vitamin B12 from plant-based food sources only. The nutrient needs (Estimated Average Requirement (EAR) or Recommended Nutrient Intake (RNI)) for four nutrients (except vitamin K) were obtained from Chinese Dietary Reference Intakes[21]. For vitamin K, only AI (adequate intake) is available and used. The results indicated that the supply of vitamins K, B6, and B9 is sufficient to meet the population's intake needs (Supplementary Table 6). Vitamin K showed low source diversity, as many raw foods in the Japanese Food Composition Database reported their nutrient content as zero. As anticipated, plant-based foods are not relevant sources of vitamins D and B12 (Supplementary Table 6). Reducing the supply of animal-based foods would further decrease the availability of vitamins D and B12 from food sources. Therefore, alternative solutions such as adequate UV exposure (for vitamin D), supplements, and food fortification are essential to ensure sufficient uptake of these nutrients, especially when shifting towards more plant-based foods. Due to the limitations of the data and differences in food composition tables, we advise caution in interpreting these findings.

Rice and wheat supply for food consumption

To estimate the rice and wheat supply for food consumption, we accounted for the refining steps and market shares of refined- and whole-grain products.

Harvested rice is initially covered with inedible husks, which are removed to produce brown rice (the conversion factor is 76%, based on Chinese national standard GB 1350-2009[38]). To obtain refined rice, widely consumed in China, the bran and germ of brown rice are removed through further refining steps[39], yielding 49.5% of the harvested rice. These conversion factors, derived from five rice types in China, were also based on GB 1350-2009[38]. However, the conversion factors may vary with different rice cultivars and the milling equipment used[38]. In this study, we differentiated rice supply into brown rice and refined rice, with annual supply proportions determined by their market shares[40] (Supplementary Fig. 2 and Supplementary Data). Similarly, the amount of refined wheat flour derived

from wheat production was 74.2%, estimated by averaging the mid-points of the reported ranges for three types of refined wheat flour in the Chinese market[41]. The allocations of wheat to refined and whole wheat flour were based on their market shares[40].

Furthermore, the reported production amount of tubers from the National Bureau of Statistics of China was manually divided by 5 to convert it from fresh weight to dry weight and make it consistent with the grain production amount[42] and match with import, export, and crop use dataset from FAOSTAT[1].

Comparison of the effects of individual measures to use nutrients more efficiently (Scenario S2)

We analyzed the individual impacts of each measure in Scenario S2. Halving food loss and waste was most effective during production, postharvest handling, and consumption (Supplementary Data, sheet 'S19'). Whole-grain substitution increased provisioning of dietary fiber, niacin, thiamin, iron, zinc, selenium. Reducing red meat intake lowered crop use for animal feed, but the reduced contributions to vitamin A, zinc, and selenium from animal-based foods were not fully offset by released food crops, causing a slight decrease in their coverage.

Impact of uncertainty in baseline coverage and source diversity due to low resolution of supply data

Due to limited data availability, some crops (e.g., vegetable subgroups, other fruits) were grouped together, despite potential variations in nutrient content across species within these groups. To assess the impact of data aggregation on coverage uncertainty, we conducted Monte Carlo simulations. In each simulation, a "food code" representing nutrient values was randomly selected for each crop within a group to serve as the representative nutrient content for that crop source. This random-selection approach was applied to crops comprising multiple species (e.g., vegetable subgroups, other cereals,

other beans, citrus fruits) or sub-products (e.g., white and yellow maize flour). For nutrients with low coverage in the baseline scenario (calcium, selenium), over 73% of the simulation results showed their coverage was still low (Extended Data Fig. 6a). For vitamin A and riboflavin, with a supply between EAR (estimated average requirement) and RNI (recommended nutrient intake) needs in the baseline scenario, over 72% and 75% of simulation results still showed supply below RNI needs (Extended Data Fig. 6b). Besides, the coverage distribution of vitamin A and vitamin C exhibited greater variation compared to other nutrients, primarily because vegetables are their main sources, and the vitamins A and C content varies across different vegetable subgroups (e.g., root vegetables, leguminous vegetables, and sprouts) (Extended Data Fig. 6). Overall, the Monte Carlo analysis indicates that the uncertainties in nutrient content arising from limitations in the details of production and trade datasets for different foods in each food group/crop group do not result in major uncertainties in which nutrients have supplies below the required needs.

The clustering of vegetables in the datasets on production and import/export prevents fully capturing the contribution of vegetables to nutrient source diversity. The best we could do to better capture the contribution of vegetables to source diversity was to disaggregate vegetables into seven subgroups based on consumption data[43]. However, this is still an underestimate of the true diversity of nutrient supply if every vegetable species was recognized as a distinct source. Hence, the reported results on source diversity are best interpreted as lower bounds, and they can be used to compare different nutrients and temporal trends. An alternative approach would be to use consumption data to split the total vegetable supply into individual species and recalculate source diversity per nutrient. Ultimately, more detailed datasets on production and import/export would be needed.

References

1. Food balance sheet. FAO. Food and Agriculture Organization of the United Nations. https://www.fao.org/faostat/. (1997–2018).

2. National data. National Bureau of Statistics of China. https://data.stats.gov.cn/. (1997–2018).

3. Ministry of Agriculture and Rural Affairs of the People's Republic of China. *China Rural Statistical Yearbook*. China Agriculture Press, Beijing (2001).

4. Zhao L, He Y, Yang Y, Yu D, Wang Z. *China nutrition and health surveillance (2010–2013): the dietary and nutritional status of Chinese population (in Chinese)*. People's Medical Publishing House (2018).

5. Huang K*, et al.* Usual Intake of Micronutrients and Prevalence of Inadequate Intake among Chinese Adults: Data from CNHS 2015–2017. *Nutrients* **14**, (2022).

6. Yu D*, et al.* Status of energy and primary nutrients intake among Chinese population in 2015–2017 (in Chinese). *Food and Nutrition in China* **27**, 5–10 (2021).

7. Wong AYS, Chan EW, Chui CSL, Sutcliffe AG, Wong ICK. The phenomenon of micronutrient deficiency among children in China: a systematic review of the literature. *Public Health Nutrition* **17**, 2605–2618 (2014).

8. Liu X*, et al.* Assessment of selenium nutritional status of school-age children from rural areas of China in 2002 and 2012. *European journal of clinical nutrition* **70**, 405–408 (2016).

9. Ayling K*, et al.* Systematic Literature Review of the Nutrient Status, Intake, and Diet Quality of Chinese Children across Different Age Groups. *Nutrients* **15**, (2023).

10. Zhao L, Ding G, Zhao W. *China Nutrition and Health Surveillance (2015–2017)*. People's Medical Publishing House (2022).

11. Han X, Ding S, Lu J, Li Y. Global, regional, and national burdens of common micronutrient deficiencies from 1990 to 2019: A secondary trend analysis based on the Global Burden of Disease 2019 study. *eClinicalMedicine* **44**, 101299 (2022).

12. Liu X*, et al.* Assessment of zinc status in school-age children from rural areas in China Nutrition and Health Survey 2002 and 2012. *Biological trace element research* **178**, 194–200 (2017).

13. Liu J, McCauley LA, Zhao Y, Zhang H, Pinto-Martin J, Group JCS. Cohort profile: The China Jintan child cohort study. *International journal of epidemiology* **39**, 668–674 (2010).

14. Liu W, Hu J, Fang Y, Wang P, Lu Y, Shen N. Vitamin D status in Mainland of China: A systematic review and meta-analysis. *EClinicalMedicine* **38**, (2021).

15. Huang Y*, et al.* Prevalence and causes of anaemia in children aged 6–23 months in rural Qinghai, China: Findings from a cross-sectional study. *BMJ open* **9**, e031021 (2019).

16. Wang J*, et al.* The influence of malnutrition and micronutrient status on anemic risk in children under 3 years old in poor areas in China. *PloS one* **10**, e0140840 (2015).

17. Tables of weight yield of food and retention factors of food constituents for the calculation of nutrient composition of cooked foods (dishes). Bognár A. Bundesforschungsanstalt für Ernährung. https://www.fao.org/uploads/media/bognar_bfe-r-02-03.pdf. (2002).

18. Standard Tables of Food Composition in Japan - 2015. Ministry of Education C, Sports, Science and Technology (Japan). https://www.mext.go.jp/en/policy/science_technology/policy/title01/detail01/1374030.htm. (2015).

19. Urbain P, Singler F, Ihorst G, Biesalski HK, Bertz H. Bioavailability of vitamin D2 from UV-B-irradiated button mushrooms in healthy adults deficient in serum 25-hydroxyvitamin D: a randomized controlled trial. *European journal of clinical nutrition* **65**, 965–971 (2011).

20. Brown EIG*, et al.* Effect of vitamin D2 supplementation on 25-hydroxyvitamin D3 status: a systematic review and meta-analysis of randomised controlled trials. *Proceedings of the Nutrition Society* **83**, (2024).

21. Chinese Nutrition Society. *Chinese DRIs Handbook (2013 Edition) (in Chinese)*, 1 edn. Standards Press of China (2014).

22. He Y*, et al.* The dietary transition and its association with cardiometabolic mortality among Chinese adults, 1982–2012: a cross-sectional population-based study. *The lancet Diabetes & endocrinology* **7**, 540–548 (2019).

23. Fang Q*, et al.* Low-opportunity-cost feed can reduce land-use-related environmental impacts by about one-third in China. *Nature food* **4**, 677–685 (2023).

24. Anti-food Waste Law of the People's Republic of China. Standing Committee of the National People's Congress of the People's Republic of China. https://www.fao.org/faolex/results/details/en/c/LEX-FAOC203240/. (2022).

25. Luo Y, Huang D, Li D, Wu L. On farm storage, storage losses and the effects of loss reduction in China. *Resources, Conservation and Recycling* **162**, 105062 (2020).

26. Milani P*, et al.* The whole grain manifesto: From Green Revolution to Grain Evolution. *Global Food Security* **34**, 100649 (2022).

27. Grain Processing Industry Development Plan (2011–2020). Ministry of Industry and Information Technology of the People's Republic of China. http://www.degradable.org.cn/policy/MIIT-125-starch.pdf. (2012).

28. Chinese Nutrition Society. *Chinese dietary guidelines (2022)*. People's Medical Publishing House (2022).

29. Schwingshackl L*, et al.* Food groups and risk of type 2 diabetes mellitus: a systematic review and meta-analysis of prospective studies. *European journal of epidemiology* **32**, 363–375 (2017).

30. Poore J, Nemecek T. Reducing food's environmental impacts through producers and consumers. *Science* **360**, 987–992 (2018).

31. Liu X*, et al.* Dietary shifts can reduce premature deaths related to particulate matter pollution in China. *Nature Food* **2**, 997–1004 (2021).

32. Peng W*, et al.* Trends in major non-communicable diseases and related risk factors in China 2002–2019: an analysis of nationally representative survey data. *The Lancet Regional Health--Western Pacific* **43**, (2024).

33. Bai Z*, et al.* China's livestock transition: Driving forces, impacts, and consequences. *Science Advances* **4**, eaar8534 (2018).

34. Harguess JM, Crespo NC, Hong MY. Strategies to reduce meat consumption: A systematic literature review of experimental studies. *Appetite* **144**, 104478 (2020).

35. Graham T, Abrahamse W. Communicating the climate impacts of meat consumption: The effect of values and message framing. *Global Environmental Change* **44**, 98–108 (2017).

36. van Zanten HHE*, et al.* Circularity in Europe strengthens the sustainability of the global food system. *Nature Food* **4**, 320–330 (2023).

37. Chinese Center for Disease Control and Prevention. *China Food Composition (in Chinese)*, 6 edn. Peking University Medical Press (2018).

38. General Administration of Quality Supervision IaQotPsR, of China. Paddy, Chinese national standard GB 1350-2009.) (2009).

39. Wu X, Guo T, Luo F, Lin Q. Brown rice: A missing nutrient-rich health food. *Food Science and Human Wellness* **12**, 1458-1470 (2023).

40. Yu D, Zhao L, Zhao W. Status and trends in consumption of grains and dietary fiber among Chinese adults (1982--2015). *Nutrition Reviews* **78**, 43-53 (2020).

41. Popular Science Handbook 2020. National Food and Strategic Reserves Administration. https://www.by.gov.cn/gzjg/qfzhggj/lszl/content/post_6523318.html. (2020).

42. Yu X, Cheng S-k, Xie G-d, Liu A-m, Lu C-x, Wang Y-y. The balance between supply and consumption of the main types of grain in China. *Journal of Natural Resources* **32**, 927-936 (2017 (in Chinese)).

43. China Health and Nutrition Survey. China Health and Nutrition Survey. https://www.cpc.unc.edu/projects/china. (2011).
