# Supplementary material for: Nutrient coverage of China’s plant-based food supply can be improved with food system adjustments
Source: Nat Food. 2026 May 1;7(5):452–63. doi: 10.1038/s43016-026-01349-6 (PMC13212153; doi:10.1038/s43016-026-01349-6)
Supplement: Supplementary file 2 — Reporting Summary [file 43016_2026_1349_MOESM2_ESM.pdf]

nature portfolio

Corresponding author(s): Wopke van der Werf, Wen-Feng Cong

Last updated by author(s): Mar 18, 2026

Reporting Summary

Nature Portfolio wishes to improve the reproducibility of the work that we publish. This form provides structure for consistency and transparency in reporting. For further information on Nature Portfolio policies, see our Editorial Policies and the Editorial Policy Checklist.

Statistics

For all statistical analyses, confirm that the following items are present in the figure legend, table legend, main text, or Methods section.

n/a	Confirmed	
☐	☒	The exact sample size (*n*) for each experimental group/condition, given as a discrete number and unit of measurement
☐	☒	A statement on whether measurements were taken from distinct samples or whether the same sample was measured repeatedly
☒	☐	The statistical test(s) used AND whether they are one- or two-sided<br>*Only common tests should be described solely by name; describe more complex techniques in the Methods section.*
☒	☐	A description of all covariates tested
☒	☐	A description of any assumptions or corrections, such as tests of normality and adjustment for multiple comparisons
☒	☐	A full description of the statistical parameters including central tendency (e.g. means) or other basic estimates (e.g. regression coefficient) AND variation (e.g. standard deviation) or associated estimates of uncertainty (e.g. confidence intervals)
☒	☐	For null hypothesis testing, the test statistic (e.g. *F*, *t*, *r*) with confidence intervals, effect sizes, degrees of freedom and *P* value noted<br>*Give P values as exact values whenever suitable.*
☒	☐	For Bayesian analysis, information on the choice of priors and Markov chain Monte Carlo settings
☒	☐	For hierarchical and complex designs, identification of the appropriate level for tests and full reporting of outcomes
☒	☐	Estimates of effect sizes (e.g. Cohen's *d*, Pearson's *r*), indicating how they were calculated

Our web collection on statistics for biologists contains articles on many of the points above.

Software and code

Policy information about availability of computer code

Data collection	No software was used for data collection. All datasets were collected manually.
Data analysis	We used Microsoft Excel to organize the input data. The calculations of coverage and source diversity in each scenario were implemented in Python 3.7.4. The code is available in Zenodo (https://doi.org/10.5281/zenodo.19036893).

For manuscripts utilizing custom algorithms or software that are central to the research but not yet described in published literature, software must be made available to editors and reviewers. We strongly encourage code deposition in a community repository (e.g. GitHub). See the Nature Portfolio guidelines for submitting code & software for further information.

Data

Policy information about availability of data

All manuscripts must include a data availability statement. This statement should provide the following information, where applicable:

- Accession codes, unique identifiers, or web links for publicly available datasets
- A description of any restrictions on data availability
- For clinical datasets or third party data, please ensure that the statement adheres to our policy

Data used in the analysis and supporting conclusions were given in the main text data availability statement. The data supporting the findings of this study, source data of figures in the main manuscript text, extended data figures, are available in the Zenodo repository (https://doi.org/10.5281/zenodo.19036893).

Research involving human participants, their data, or biological material

Policy information about studies with human participants or human data. See also policy information about sex, gender (identity/presentation), and sexual orientation and race, ethnicity and racism.

Reporting on sex and gender	We used the term sex within the paper. When calculating dietary nutrient needs, we used sex proportions at the national level and the recommended nutrient intakes, both of which have already been reported for all the age and sex groups.
Reporting on race, ethnicity, or other socially relevant groupings	We do not use any socially constructed or socially relevant categorization variables in this research.
Population characteristics	We do not have any human research participants.
Recruitment	The data used in this paper are from public sources and existing publications. There was no need to recruit participants. There is no risk of selection bias.
Ethics oversight	The data used in this paper are from public sources and existing publications. There was no need to obain approval for the protocol.

Note that full information on the approval of the study protocol must also be provided in the manuscript.

Field-specific reporting

Please select the one below that is the best fit for your research. If you are not sure, read the appropriate sections before making your selection.

☐ Life sciences ☐ Behavioural & social sciences ☒ Ecological, evolutionary & environmental sciences

For a reference copy of the document with all sections, see nature.com/documents/nr-reporting-summary-flat.pdf

Ecological, evolutionary & environmental sciences study design

All studies must disclose on these points even when the disclosure is negative.

Study description	This study evaluates the potential of China's plant-based food supply (PFS) to sufficiently and diversely meet population-level intake needs for energy and 17 nutrients. Using data from 1997 to 2018 in China, we constructed bipartite networks at national and provincial levels per year, linking food crops, animal-based food sources, and 17 nutrients along with dietary energy. We quantified each nutrient's supply/demand ratio (coverage) and source diversity. Then, we analyzed the uncertainties of coverage for each nutrient in the baseline Scenario (S1) by implementing the Monte Carlo simulations. Finally, we analyzed the impact of two alternative strategies on nutrient coverage and source diversity. Scenario S2 aims to use the nutrients from PFS more efficiently by reducing direct nutrient losses (Scenario S2a: reduce food loss and waste; and use more whole grains) and also reducing indirect nutrient losses (Scenario S2b: reduce red meat intake to free up crops for direct human consumption rather than using them as animal feed). Scenario S3 (Self-sufficiency) aims to analyze which level of self-sufficiency can be reached when all domestically harvested food crops are used for direct human consumption and international trade is absent. The analysis is done at the national (Scenario S3a) and provincial levels, i.e., without inter-provincial trade (Scenario S3b).
Research sample	The nutrient content of one random member of each food group.
Sampling strategy	For the sampling strategy in the Monte Carlo simulations, a "food code" representing the nutrient values of each crop was randomly selected in each iteration to serve as the representative nutrient content for that crop source. The "random.choice" function from the random package of Python was used.
Data collection	The data are retrieved from publicly available datasets, literature, or upon request from the authors in the existing literature.
Timing and spatial scale	The data on domestic production, population, age, and sex groups were collected annually from 1997 to 2018 at the provincial-level in China. For the analysis at the national level, the provincial production data were added up to get the total production within the country. The international trade and crop use datasets were collected annually from 1997 to 2018. The other datasets are retrieved from the literature that implemented analysis within the studied period.
Data exclusions	We do not exclude any data from our analysis.
Reproducibility	Our findings are replicable using the described methods and datasets.
Randomization	For the Monte Carlo simulations, we employed the "random.choice" function from Python's random module, using a fixed random seed of 42 to ensure reproducibility.
Blinding	Blinding is not relevant to our study because all analyses were based on secondary data sources.

Did the study involve field work? ☐ Yes ☒ No

Reporting for specific materials, systems and methods

We require information from authors about some types of materials, experimental systems and methods used in many studies. Here, indicate whether each material, system or method listed is relevant to your study. If you are not sure if a list item applies to your research, read the appropriate section before selecting a response.

Materials & experimental systems

n/a	Involved in the study
☒	☐ Antibodies
☒	☐ Eukaryotic cell lines
☒	☐ Palaeontology and archaeology
☒	☐ Animals and other organisms
☒	☐ Clinical data
☒	☐ Dual use research of concern
☒	☐ Plants

Methods

n/a	Involved in the study
☒	☐ ChIP-seq
☒	☐ Flow cytometry
☒	☐ MRI-based neuroimaging

Plants

Seed stocks	Not applicable to this study.
Novel plant genotypes	Not applicable to this study.
Authentication	Not applicable to this study.
